# The m^5^C reader Ybx1 regulates embryonic cortical neurogenesis by promoting progenitor cell cycle progression

**DOI:** 10.1371/journal.pbio.3003175

**Published:** 2025-05-28

**Authors:** Jian Zhang, Pengfei Che, Zhuoxuan Yang, Pingrui Zhang, Yuxuan Shui, Xibin Lu, Jiuzhou Xu, Yuanchu She, Yanbo Zhang, Jun Yu, Sheng-Jian Ji

**Affiliations:** 1 Department of Neuroscience, School of Life Sciences, Southern University of Science and Technology, Shenzhen, Guangdong, China; 2 The Core Research Facilities, Southern University of Science and Technology, Shenzhen, Guangdong, China; Inserm U1208, FRANCE

## Abstract

The reversible epitranscriptomic mark, 5-methylcytosine (m^5^C) modification, is implicated in numerous cellular processes, but its role in neural development remains largely unexplored. In this study, we discovered high expression of the m^5^C reader Ybx1 in the developing mouse cortex. To elucidate its role in cortical development, *Ybx1* was ablated in embryonic cortical neural stem cells (NSCs). Interestingly, conditional knockout (cKO) of *Ybx1* led to perinatal mortality in mice, along with abnormal cortical development. Cortical progenitor cells lacking *Ybx1* exhibited impaired proliferation and differentiation. Multi-omics analysis identified the target mRNAs of Ybx1, which encode the key cell cycle regulatory proteins converging on cyclin D2 (Ccnd2). Ybx1 was found to regulate the stability of its target transcripts. Both knockdown and overexpression of Ybx1 targets via in utero electroporation confirmed that they mediated Ybx1 regulation of proliferation and differentiation of neural precursor cells. Further analysis showed that the G1 to S phase transition in cortical progenitor cells is delayed in the *Ybx1* cKO. This study highlights the crucial function of the m^5^C reader protein Ybx1 in promoting cell cycle progression of the embryonic cortical progenitors, essential for proper cortical development.

## Abstract 2

5-methylcytosine (m5C) RNA modifications are implicated in various cellular functions, but their role during neural development is poorly understood. This study shows that the m5C reader protein Ybx1 plays a crucial role in promoting the cell cycle progression of embryonic cortical progenitor cells, essential for proper cortical development.

## Introduction

Mammalian cerebral cortex development represents a highly regulated process, encompassing diverse regulatory mechanisms at genetic, molecular, and cellular levels [1–3]. At the onset of brain development, the cortex is populated by neural epithelial progenitor (NEP) cells that span from the ventricle (apical) to the surface (basal) of the neural tube, undergoing symmetric divisions to augment the progenitor pool [4–6]. Subsequently, these NEP cells undergo a transition into apical radial glial cells (RGCs). RGCs are capable of self-renewal, and can either differentiate directly into neurons or produce secondary basal progenitors (BPs) which are also known as the intermediate progenitors (IPs) [7,8]. The apical progenitor cells (APs) in the ventricular zone (VZ) and the IPs in the subventricular zone (SVZ) have the ability to differentiate into various neuronal subtypes. The proliferation and differentiation of neural stem cells (NSCs) are governed by highly intricate and finely orchestrated regulatory mechanisms. Any disruption in these regulatory processes has the potential to cause abnormalities in cortical development [9,10].

An increasing body of research underscores the significant role of post-transcriptional RNA modifications in brain development and function [10,11]. To date, over 150 distinct post-transcriptional modifications have been identified. High-throughput sequencing methods have revealed the dynamic “epitranscriptome” landscape of numerous mRNA modifications in various organisms, including *N*^6^-methyladenosine (m^6^A), 5-methylcytosine (m^5^C), *N*^1^-methyladenosine (m^1^A) and pseudouridine (ψ) [12]. Our lab has a keen interest in exploring the role of RNA modifications in the development of the nervous system. We have previously investigated the regulatory roles of m^6^A modification in various regions, developmental processes, and functions of the nervous system, as well as its involvement in neurological disorders [13–18].

Among the numerous RNA modifications, 5-methylcytosine (m^5^C), as one of the most abundant RNA modifications in eukaryotic cells, is gradually recognized as a crucial form of RNA metabolic regulation. Initially, it was believed that this RNA modification predominantly occurred on rRNA and tRNA. However, with advances in biochemical detection technologies, the transcriptome-wide mapping of m^5^C has revealed that most m^5^C sites are also present in mRNAs, and are located near translation initiation sites and 3′-untranslated regions (3′ UTRs), indicating the role of m^5^C modification in regulating mRNA translation and stability [19,20]. The dynamic profiling of methylated mRNAs in the developing brain [21], implies potential roles of m^5^C in neuronal development. RNA modifications could regulate RNA processing by recruiting specific “reader” proteins, which recognize and bind their target transcripts and mediate functions of modifications on RNAs. Thus, to elucidate the roles of m^5^C modification in neuronal development, it is crucial to identify and characterize the m^5^C reader proteins in nervous system.

Recent studies have revealed that Y-box binding protein 1 (Ybx1) can recognize m^5^C-modified mRNA by an indole ring within its cold shock domain, playing a pivotal role in regulating RNA metabolism [22,23]. Subsequently, Ybx1 has been confirmed as an m^5^C “reader” in various systems. In zebrafish embryos, Ybx1 primarily recognizes m^5^C-modified mRNA through its cold shock domain (CSD). It recruits the protein Pabpc1a to methylated transcripts to prevent the decay of maternal mRNA, thereby facilitating the transition from maternal to zygotic gene expression [24,25]. In Drosophila, studies suggest that YPS, a homolog closely related to human YBX1, exhibits a preferential binding to m^5^C-enriched mRNA within germline cells. This binding is crucial for sustaining the proliferation and differentiation of germline stem cells in the ovaries [23]. In mice, global knockout of *Ybx1* gene results in early embryonic lethality [26,27]. However, the role of Ybx1 as the m^5^C reader in mammalian neuronal development remains poorly understood.

Here, we observed high expression of Ybx1 in the mouse embryonic cortex. Analysis using conditional knockout (cKO) of *Ybx1* in the nervous system and neurosphere culture experiments revealed the essential role of Ybx1 in NSC self-renewal. Further mechanistic exploration identified its target mRNAs which mediate the function of Ybx1 in regulating cell cycle progression in neural progenitor cells. Together, our study elucidates the vital role of the m^5^C reader Ybx1 in the cortical progenitors, thereby offering novel insights into the epitranscriptomic mechanisms governing mammalian cerebral cortex development.

## Results

### Conditional knockout of *Ybx1* in the nervous system results in perinatal lethality and impaired cortical development

**Fig 1:**
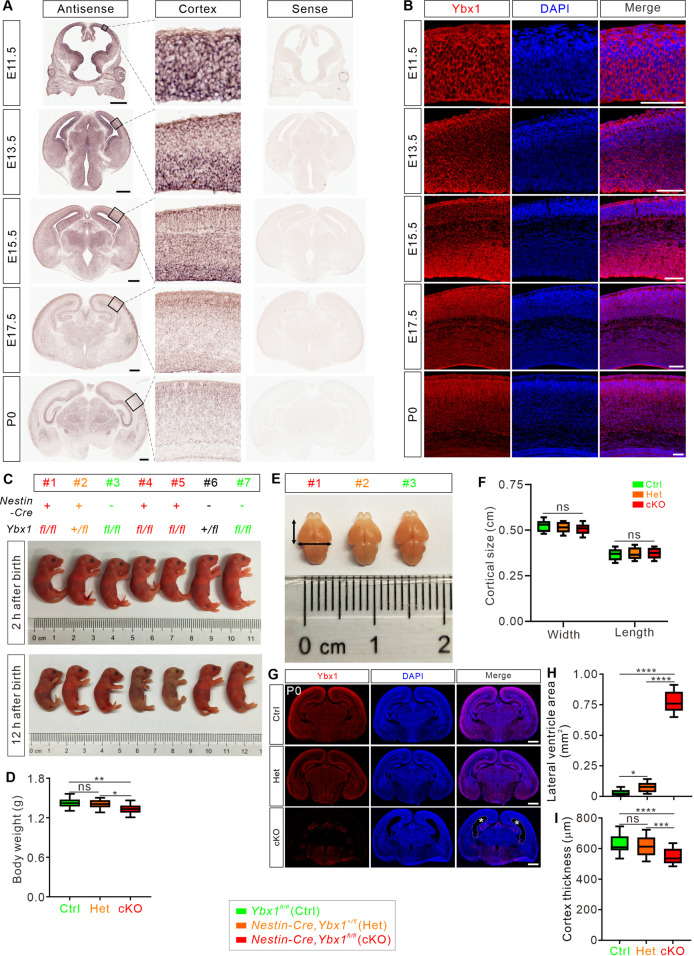
Ybx1 is highly expressed in the progenitor cells of earlier embryonic mouse cortex and conditional ablation of *Ybx1* causes impaired cortical development. **(A)** In situ hybridization using DIG-labeled RNA probes (antisense and sense) targeting *Ybx1* was performed on coronal sections of mouse brains at E11.5 to P0. Representative cortical regions were shown as magnified images. Scale bars, 500 μm. **(B)** Immunostaining for Ybx1 protein was done on coronal sections of mouse brains at E11.5, E13.5, E15.5, E17.5, and P0. Representative cortical regions were shown. Scale bars, 100 μm. **(C)** Representative images of P0 neonatal mice from the same litter with different genotypes were shown at 2 and 12 h after birth, respectively. **(D)** Quantitative analysis of neonatal body weight at P0 was shown as box and whisker plots. *Ybx1*^*fl/fl*^, *Nestin-Cre, Ybx1*^*+/fl*^*, and Nestin-Cre, Ybx1*^*fl/fl*^ were designated as control (Ctrl), heterozygous (Het), and cKO, respectively. Ctrl (*n* = 39 pups) vs. Het (*n* = 26 pups), *p* = 0.37; Ctrl vs. cKO (*n* = 28 pups), **p = 0.0014; Het vs. cKO, *p = 0.021; ns, not significant; all by one-way ANOVA followed by Tukey’s multiple comparison test. **(E)** Representative images of the P0 brains from cKO (#1), Het (#2), and Ctrl (#3) pups shown in **(D)**. Black arrowed lines indicate the cortical length and width. **(F)** Quantitative analysis of cortical length and width at P0 was shown as box and whisker plots. *n* = 21 pups for Ctrl, *n* = 15 pups for Het, *n* = 16 pups for cKO: *p* = 0.65 for width, *p* = 0.80 for length; ns, not significant; all by one-way ANOVA followed by Tukey’s multiple comparison test. **(G)** Representative images of P0 coronal brain sections stained with an antibody against Ybx1 protein demonstrated efficient ablation of Ybx1 in the cKO brain. The asterisk indicates the enlarged ventricle in the cKO brain. Scale bar, 1,000 μm. **(H)** Quantification of lateral ventricle area of P0 coronal brain sections was presented as box and whisker plots: Ctrl (*n* = 21 confocal fields) vs. Het (*n* = 22 confocal fields), *p = 0.035; Ctrl vs. cKO (*n* = 21 confocal fields), ****p = 2.03E−11; Het vs. cKO, ****p = 2.03E−011; all by one-way ANOVA followed by Tukey’s multiple comparison test. **(I)** Quantification of P0 cortex thickness was presented as box and whisker plots: Ctrl (*n* = 20 confocal fields) vs. cKO (*n* = 21 confocal fields), ****p = 6.73-05; Het (*n* = 19 confocal fields) vs. cKO, **p = 0.0014; ns, not significant; all by one-way ANOVA followed by Tukey’s multiple comparison test. The data underlying all the graphs shown in the figure are included in S1 Data.

To investigate the role of the m^5^C reader Ybx1 in brain development, we first examined its spatiotemporal expression patterns within the developing mouse brains. In situ hybridization and immunofluorescence assays showed that Ybx1 is highly expressed in the whole cortex from E11.5 to E15.5 (Fig 1A and 1B). To further confirm the expression pattern of Ybx1 protein in the cortex regions at E13.5 and E15.5, we performed immunofluorescence of Ybx1 using adjacent sections and co-stained with markers of radial glial cells (RGCs, marked by Pax6) and intermediate progenitors (IPs, marked by Tbr2). The results showed that Ybx1 exhibits high expression levels in both progenitor cells and neurons at E13.5 and E15.5 (S1A Fig), which is consistent with the published single-cell RNA sequencing (scRNA-seq) data [28]. As cortical neurogenesis completes at E17.5 and postnatal day 0 (P0), Ybx1 expression diminishes in the proliferative zones but remains prominent in the cortical plate (CP) (Fig 1A and 1B). In summary, the high expression of Ybx1 in the embryonic mouse cortex suggests its potential role in the embryonic cortical neurogenesis.

To explore the potential functions of Ybx1 during the onset of neurogenesis, we generated mice with floxed *Ybx1* alleles (referred to as *Ybx1*^*fl/fl*^) by crossing them with *Nestin-Cre* mice (S1B Fig). We analyzed three genotypes: *Nestin-Cre,Ybx1*^*fl/fl*^ (*Ybx1* cKO); *Nestin-Cre,Ybx1*^*+/fl*^ (heterozygote, Het); and *Ybx1*^*fl/fl*^ (control, Ctrl) mice. All *Ybx1* cKO mice died within 1–2 days after birth, with no surviving pups observed after 2 days. *Ybx1* cKO mice are smaller and have reduced body weight compared to their control littermates at postnatal day 0 (P0) (Fig 1C and 1D). When examining the brains of P0 cKO pups, no obvious anatomical abnormalities were observed, and there were no differences in cortical length and width compared to control littermates (Fig 1E and 1F). Subsequent immunostaining of coronal brain slices from P0 mice confirmed the successful ablation of Ybx1 in the cKO cortex (Fig 1G). However, we noted a significant enlargement of the lateral ventricles and a decrease in cortical thickness in *Ybx1* cKO pups, compared to their control littermates (Fig 1G–1I and S1C Fig). These data suggest that the removal of *Ybx1* during the initiation of neurogenesis leads to defects in cortical development.

### Conditional ablation of *Ybx1* leads to a reduction in cortical progenitor cell pool and a decrease in cortical thickness

**Fig 2:**
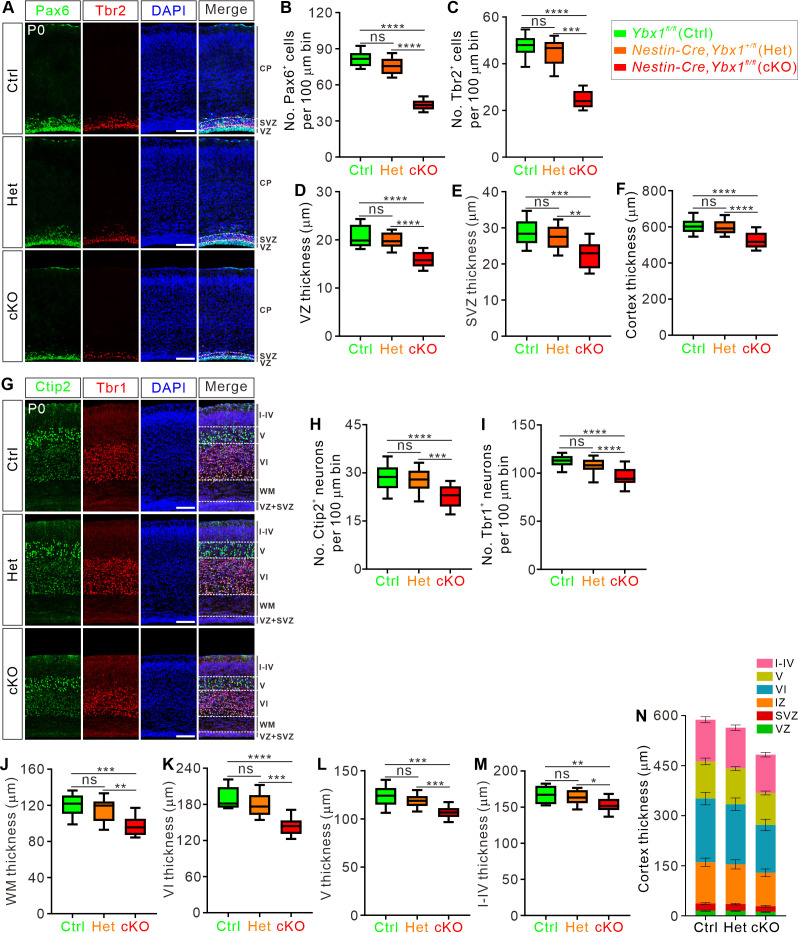
Conditional ablation of *Ybx1* leads to a reduction in cortical progenitor cell pool and a decrease in cortical thickness. **(A)** Immunostaining of coronal brain sections at P0 using antibodies against radial glial progenitor marker Pax6 and intermediate progenitor marker Tbr2. Representative cortical regions were shown. White dotted lines mark the boundaries. Scale bar, 100 μm. **(B-F)** Quantification of Pax6^+^ **(B)** and Tbr2^+^ **(C)** cell numbers, and cortical layer thicknesses **(D–F)** shown in **(A)**. All statistical data are presented as box and whisker plots. For Pax6^+^ cell numbers, Ctrl (*n* = 26 confocal fields) vs. cKO (*n* = 24 confocal fields), ****p = 1.42E−05; Het (*n* = 23 confocal fields) vs. cKO, ****p = 9.54E−05. For Tbr2^+^ cell numbers, Ctrl (*n* = 26 confocal fields) vs. cKO (*n* = 24 confocal fields), ****p = 1.19E−05; Het (*n* = 23 confocal fields) vs. cKO, ***p = 1.85E−04. For VZ thickness, Ctrl (*n* = 26 confocal fields) vs. cKO (*n* = 24 confocal fields), ****p = 9.26E−05; Het (*n* = 23 confocal fields) vs. cKO, ****p = 1.33E−05. For SVZ thickness, Ctrl (*n* = 26 confocal fields) vs. cKO (*n* = 24 confocal fields), ***p = 9.49E−04; Het (*n* = 23 confocal fields) vs. cKO, **p = 0.0077. For whole cortical thickness, Ctrl (*n* = 26 confocal fields) vs. cKO (*n* = 24 confocal fields), ****p = 8.47E−06; Het (*n* = 23 confocal fields) vs. cKO, ****p = 2.61E−05. **(G)** Immunostaining of coronal brain sections at P0 for layer V marker Ctip2 and layer VI marker Tbr1. Representative cortical regions were shown. White dotted lines mark the boundaries of each layer. Scale bar, 100 μm. **(H–M)** Quantification of Ctip2^+^ **(H)** and Tbr1^+^ **(I)** neuron numbers, and the thickness of WM layer **(J)**, layer VI **(K)**, layer V **(L)**, and layers I–IV **(M)** shown in **(G)**. All statistical data are presented as box and whisker plots. For Ctip2^+^ neuron numbers, Ctrl (*n* = 22 confocal fields) vs. cKO (*n* = 20 confocal fields), ****p = 1.89E−06; Het (*n* = 20 confocal fields) vs. cKO, ***p = 1.62E−04. For Tbr1^+^ neuron numbers, Ctrl (*n* = 19 confocal fields) vs. cKO (*n* = 21 confocal fields), ****P = 4.62E−08; Het (*n* = 21 confocal fields) vs. cKO, ****P = 5.50E−05. For WM layer thickness, Ctrl (*n* = 22 confocal fields) vs. cKO (*n* = 20 confocal fields), ***p = 3.50E−04; Het (*n* = 20 confocal fields) vs. cKO, **p = 0.0038. For layer VI thickness, Ctrl (*n* = 22 confocal fields) vs. cKO (*n* = 20 confocal fields), ****p = 1.43E−05; Het (*n* = 20 confocal fields) vs. cKO, ***p = 2.56E−04. For layer V thickness, Ctrl (*n* = 22 confocal fields) vs. cKO (*n* = 20 confocal fields), ***p = 5.16E−04; Het (*n* = 20 confocal fields) vs. cKO, ***p = 5.71E−04. For thickness of layers, I–IV, Ctrl (*n* = 22 confocal fields) vs. cKO (*n* = 20 confocal fields), **p = 0.0093; Het (*n* = 20 confocal fields) vs. cKO, *p = 0.032. **(N)** A stacked bar chart summarizing the data in **(D–F, J–M)** shows the distribution of each layer in P0 cortex. At least 3 mice were analyzed for each genotype. All analyses were performed by one-way ANOVA followed by Tukey’s multiple comparison test. ns, not significant. The data underlying all the graphs shown in the figure are included in S1 Data.

Within the developing neocortex, two distinct progenitor cell types are present. Radial glial cells (RGCs) situated in the ventricular zone (VZ) can be marked by Pax6. Proliferative populations derived from RGCs form the subventricular zone (SVZ) and are designated as intermediate progenitor cells (IPCs). The transcription factor Tbr2 is primarily expressed in IPCs within the SVZ [7]. To more comprehensively characterize the impact of *Ybx1* deletion on cortical development, we performed immunostaining for Pax6 and Tbr2 to assess the neural progenitor cell pool (Fig 2A). The number of Pax6^+^ RGCs was reduced by over 50% in P0 *Ybx1* cKO mice compared to control littermates, while Tbr2^+^ cells decreased by 30% (Fig 2B and 2C). Consistently, we observed that the thicknesses of both VZ and SVZ were significantly reduced in *Ybx1* cKO pups compared to the control ones (Fig 2D and 2E), and the overall thickness of the cortex decreased by 20% (Fig 2F). All experimental results in this study (including the following figures) showed no significant differences between the heterozygous and control littermates.

Next we investigated whether the decrease in the neural stem cell pool due to Ybx1 loss was caused by apoptosis. TUNEL staining in the cerebral cortex showed that the number of apoptotic cells did not change in P0 *Ybx1* cKO brain cortex compared to the control group (S2A and S2B Fig). Subsequently, we sought to investigate whether apoptosis starts at earlier stages. We performed immunofluorescence for cleaved caspase-3 (CC3) on cortical tissue at E13.5 and E15.5 (S2C and S2E Fig). Consistent with the data from the P0 stage, staining for CC3 revealed that loss of Ybx1 does not result in an increase in the number of apoptotic cells at E13.5 or E15.5 (S2C–S2F Fig). These results suggest that the absence of Ybx1 reduces the number of progenitor cells in the developing neocortex, which is not mediated by apoptosis.

Next, we assessed the impact of Ybx1 loss on cortical neurogenesis. In P0 mice, neurons differentiated from RGCs exist in six different cortical layers, with early-born neurons occupying the deep layers of the neocortex and later-born neurons migrating to the superficial layers, forming a normative six-layered structure in an “inside-out” manner [29]. To investigate the effect of Ybx1 loss-induced depletion of neural stem cells on the generation of neurons in different cortical layers, we conducted immunostaining of Tbr1 and Ctip2 at P0, which label cortical layers VI and V, respectively. This allowed us to distinguish layers I–IV, V, VI, and white matter (WM) (Fig 2G). The numbers of Ctip2^+^ layer V neurons and Tbr1^+^ layer VI neurons are decreased in P0 *Ybx1* cKO mice compared with the control mice (Fig 2H and 2I). Although the overall laminar structure is similar between *Ybx1* cKO and control mice (Fig 2G), each layer in *Ybx1* cKO mice exhibits varying degrees of thickness reduction (Fig 2J–2N). These results indicate that the loss of Ybx1 impairs cortical neurogenesis.

### Ybx1 is required for maintenance of the cortical progenitor pool from E13.5

**Fig 3:**
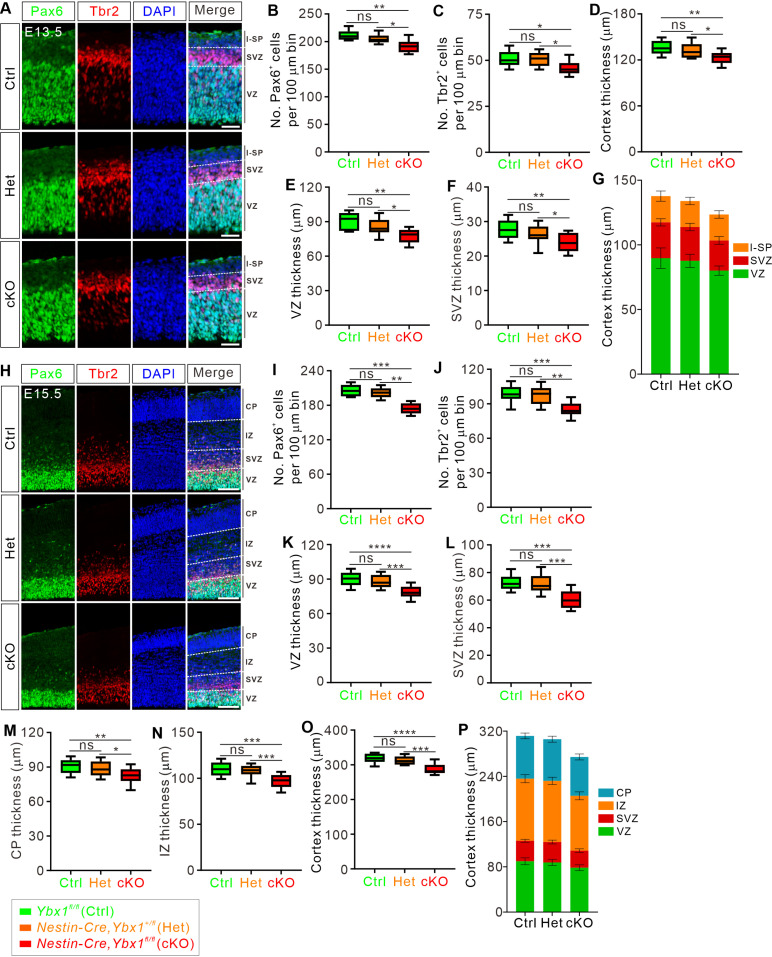
Ybx1 is required for maintenance of the cortical progenitor pool from E13.5. **(A)** E13.5 coronal brain sections were immunostained with antibodies against the radial glial cell marker Pax6 and the intermediate progenitor marker Tbr2. Representative cortical regions were shown. White dotted lines mark the boundaries. Scale bar: 50 μm. **(B–F)** Quantification of Pax6^+^ **(B)** and Tbr2^+^ **(C)** cell numbers, and cortical layer thicknesses **(D to F)** shown in **(A)**. All statistical data are presented as box and whisker plots. For Pax6, Ctrl (*n* = 21 confocal fields) vs. cKO (*n* = 22 confocal fields), **p = 0.0018; Het (*n* = 18 confocal fields) vs. cKO, *p = 0.019. For Tbr2, Ctrl (*n* = 21 confocal fields) vs. cKO (*n* = 22 confocal fields), *p = 0.025; Het (*n* = 18 confocal fields) vs. cKO, *p = 0.014. For whole cortical thickness, Ctrl (*n* = 21 confocal fields) vs. cKO (*n* = 22 confocal fields), **p = 0.0018; Het (*n* = 18 confocal fields) vs. cKO, *p = 0.016. For VZ thickness, Ctrl (*n* = 21 confocal fields) vs. cKO (*n* = 22 confocal fields), **p = 0.0023; Het (*n* = 18 confocal fields) vs. cKO, *p = 0.026. For SVZ thickness, Ctrl (*n* = 21 confocal fields) vs. cKO (*n* = 22 confocal fields), **p = 0.0055; Het (*n* = 18 confocal fields) vs. cKO, *p = 0.047. **(G)** A stacked bar chart summarizing the data in **(D–F)** shows the distribution of each layer in E13.5 cortex. **(H)** E15.5 coronal brain sections were immunostained with antibodies against the radial glial cell marker Pax6 and the intermediate progenitor marker Tbr2. Representative cortical regions were shown. White dotted lines mark the boundaries. Scale bar: 100 μm. **(I–O)** Quantification of Pax6^+^ **(I)** and Tbr2^+^ **(J)** cell numbers, and cortical layer thicknesses **(K–O)** shown in **(H)**. All statistical data are presented as box and whisker plots. For Pax6, Ctrl (*n* = 24 confocal fields) vs. cKO (*n* = 22 confocal fields), ***p = 2.93E−04; Het (*n* = 21 confocal fields) vs. cKO, **p = 0.0071. For Tbr2, Ctrl (*n* = 24 confocal fields) vs. cKO (*n* = 22 confocal fields), ***p = 8.58E−04; Het (*n* = 21 confocal fields) vs. cKO, **p = 0.0042. For VZ thickness, Ctrl (*n* = 24 confocal fields) vs. cKO (*n* = 22 confocal fields), ****p = 5.60E−05; Het (*n* = 21 confocal fields) vs. cKO, ***p = 2.72E−04. For SVZ thickness, Ctrl (*n* = 24 confocal fields) vs. cKO (*n* = 22 confocal fields), ***p = 2.75E−04; Het (*n* = 21 confocal fields) vs. cKO, ***p = 8.94E−04. For CP thickness, Ctrl (*n* = 24 confocal fields) vs. cKO (*n* = 22 confocal fields), **p = 0.0062; Het (*n* = 21 confocal fields) vs. cKO, *p = 0.034. For IZ thickness, Ctrl (*n* = 24 confocal fields) vs. cKO (*n* = 22 confocal fields), ***p = 1.99E−04; Het (*n* = 24 confocal fields) vs. cKO, ***p = 5.31E−04. For whole cortical thickness, Ctrl (*n* = 24 confocal fields) vs. cKO (*n* = 22 confocal fields), ****p = 1.73E−05; Het (*n* = 21 confocal fields) vs. cKO, ***p = 1.68E−04. **(P)** A stacked bar chart summarizing the data in **(K–O)** shows the distribution of each layer in E15.5 cortex. At least 3 embryos were analyzed for each genotype. All analyses were performed by one-way ANOVA followed by Tukey’s multiple comparison test. ns, not significant. The data underlying all the graphs shown in the figure are included in S1 Data.

To determine at which embryonic stage the reduction in the NSC pool occurs in the cKO mice, we first checked the knockout efficiency of Ybx1 in E11.5, E13.5, E15.5, and E17.5 cortex. Immunostaining showed that the Ybx1 signal in the cKO cortex was nearly absent starting from E13.5 onwards (S3A Fig). Ybx1 signals in the heterozygous mice were comparable to those in control mice at all stages studied. Pax6 immunostaining on cortical sections of E11.5 embryos showed no significant changes in cortical thickness and progenitor cell pools in *Ybx1* cKO compared to control littermates (S3B–S3D Fig), which is consistent with the fact Ybx1 was not ablated yet at E11.5 (S3A and S3B Fig). By E13.5, the numbers of Pax6^+^ and Tbr2^+^ cells in the *Ybx1* cKO cortex diminished by 11.6% and 10.1%, respectively, in comparison to control embryos (Fig 3A–3C). Meanwhile, we observed a slight decrease in the thickness of the VZ, SVZ, and whole cortex at E13.5 (Fig 3D–3G). At E15.5, the reduction in the numbers of Pax6^+^ and Tbr2^+^ cells in *Ybx1* cKO cortex became more pronounced, counting as 18.4% and 13.1%, respectively (Fig 3H–3J). Significant decreases in the thickness of each layer were also observed in the cKO embryos (Fig 3K–3P). Similarly pronounced reductions were observed in E17.5 *Ybx1* cKO brains (S3E–S3N Fig).

These findings suggest that loss of the *Ybx1* leads to a reduction in the progenitor cell pool in the mouse cortex from E13.5, followed by decreases in thickness of cortical layers composed of late-born neurons.

### Ybx1 regulates proliferation and differentiation of cortical progenitor cells

**Fig 4:**
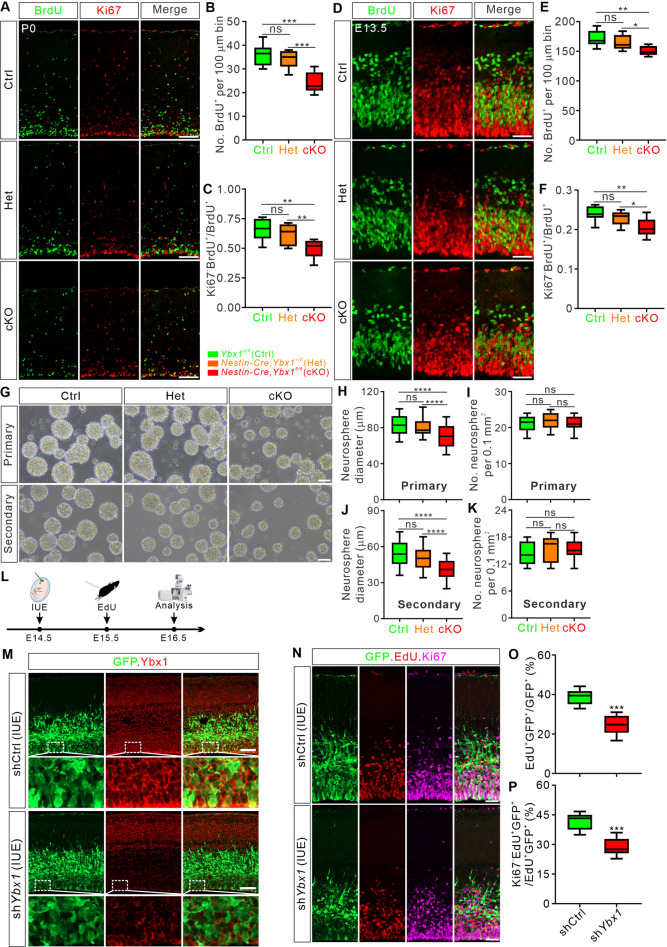
Ybx1 regulates proliferation and differentiation of cortical progenitor cells. **(A)** Coronal brain sections at P0 were stained with antibodies recognizing BrdU and Ki67. Pregnant mothers received a BrdU pulse 24 h before pup dissection at P0. Representative cortical regions were shown. Scale bar: 100 μm. **(B and C)** Quantification of BrdU^+^ cell numbers **(B)** and the percentage of cells exiting the cell cycle **(C)** at P0 shown in **(A)**. All statistical data are presented as box and whisker plots. For BrdU^+^ cell numbers, Ctrl (*n* = 27 confocal fields) vs. cKO (*n* = 24 confocal fields), ***p = 2.11E−04; Het (*n* = 23 confocal fields) vs. cKO, ***p = 4.24E−04. For Ki67^−^BrdU^+^/BrdU^+^, Ctrl (*n* = 27 confocal fields) vs. cKO (*n* = 24 confocal fields), **p = 0.0015; Het (*n* = 23 confocal fields) vs. cKO, **p = 0.0051. **(D)** Coronal brain sections at E13.5 were stained with antibodies recognizing BrdU and Ki67. Pregnant mothers received a BrdU pulse 24 h before embryo collection. Representative cortical regions were shown. Scale bar, 50 μm. **(E and F)** Quantification of BrdU^+^ cell numbers **(E)** and the percentage of cells exiting the cell cycle **(F)** at E13.5 shown in **(D)**. All statistical data are presented as box and whisker plots. For BrdU^+^ cell numbers, Ctrl (*n* = 22 confocal fields) vs. cKO (*n* = 25 confocal fields), **p = 0.0052; Het (*n* = 23 confocal fields) vs. cKO, *p = 0.012. For Ki67^−^BrdU^+^/BrdU^+^, Ctrl (*n* = 22 confocal fields) vs. cKO (*n* = 25 confocal fields), **p = 0.0028; Het (*n* = 23 confocal fields) vs. cKO, *p = 0.027. **(G)** Representative images of primary and secondary neurospheres formed by NSCs isolated at E13.5. Scale bar, 50 μm. **(H–K)** Quantification of the sizes **(H, J)** and numbers **(I, K)** of the primary **(H, I)** and secondary **(J, K)** neurospheres show in **(G)**. All statistical data are presented as box and whisker plots. In **H**, Ctrl (*n* = 42 neurospheres) vs. cKO (*n* = 38 neurospheres), ****p = 6.65E−05; Het (*n* = 46 neurospheres) vs. cKO, ****p = 5.22E−05. In **J**, Ctrl (*n* = 42 neurospheres) vs. cKO (*n* = 38 neurospheres), ****p = 2.76E−05; Het (*n* = 46 neurospheres) vs. cKO, ****p = 2.26E−05. **(L)** Schematic drawings of the IUE experiment. Pregnant mice were injected at E14.5 with plasmids. Subsequently, a single EdU pulse was administrated at E15.5, and embryos were dissected at E16.5 for analysis. **(M)** Confirmation of Ybx1 knockdown in the cortex after IUE of sh*Ybx1*. Immunostaining for GFP and Ybx1 on coronal sections of E16.5 cortex. Representative cortical regions were shown. Scale bar, 50 μm. **(N)** Immunostaining for GFP, EdU, and Ki67 on coronal sections of E16.5 mouse cortex after knockdown of Ybx1 using IUE. Representative cortical regions were shown. Scale bar, 50 μm. **(O and P)** Quantification of percentage of EdU^+^GFP^+^/GFP^+^ **(O)** and Ki67^-^EdU^+^/EdU^+^GFP^+^ **(P)** shown in **(N)**. Data are presented as box and whisker plots: in **O**, shCtrl (*n* = 22 confocal fields) vs. sh*Ybx1* (*n* = 19 confocal fields), ***p = 1.38E−04; in **P**, shCtrl (*n* = 22 confocal fields) vs. sh*Ybx1* (*n* = 19 confocal fields), ***p = 8.30E−04. At least 3 pups or embryos were analyzed for each genotype or condition. Analyses were performed by one-way ANOVA followed by Tukey’s multiple comparison test **(B, C; E, F; H–**K), or by unpaired Student *t* test **(O, P)**. ns, not significant. The data underlying all the graphs shown in the figure are included in S1 Data.

To investigate the mechanism underlying the observed reduction in cortical NSCs due to Ybx1 loss, we performed in vivo BrdU pulse labeling experiments in mice. Pregnant mice at E19.5 received BrdU injections, and 24 h post-injection, coronal brain sections from P0 pups were prepared for BrdU and Ki67 immunostaining analysis. BrdU incorporation can be used to monitor the proliferation. We observed a significant decrease in the cumulative number of BrdU-labeled cells in the cKO cortex compared to control cortex at P0 (Fig 4A and 4B), suggesting that proliferation may be inhibited in *Ybx1* cKO embryos or the neural progenitor cell pool of *Ybx1* cKO embryos is simply reduced at P0. Ki67, a cell cycle marker closely associated with cell proliferation, allows for the assessment of cell cycle exit rates, as indicated by the proportion of Ki67^−^BrdU^+^ in total BrdU^+^ cells. Immunofluorescence results showed that, relative to the littermate controls, the proportion of cells exiting the cell cycle (Ki67^−^BrdU^+^/BrdU^+^) was significantly reduced in *Ybx1* cKO embryos, indicating that the ablation of *Ybx1* also inhibited the differentiation of cortical NSCs (Fig 4A and 4C).

We further backtracked the earlier embryonic stages at E13.5, E15.5, and E17.5 using BrdU/Ki67 staining analysis. From E13.5 onwards, the total number of BrdU-labeled cells and the proportion of cells exiting the cell cycle in the *Ybx1* cKO cortex were reduced, in comparison to control cortex (Fig 4D–4F). Similar experimental results were obtained in E15.5 and E17.5 cKO cortex (S4A–S4F Fig). Together, these results suggest that Ybx1 is required for normal proliferation and differentiation of cortical progenitor cells.

To further understand the impact of Ybx1 on the self-renewal of neural stem cells, we performed in vitro neurosphere assay using dissociated cortical NSCs from E13.5 embryos. While neural stem cells from *Ybx1* cKO, heterozygous, and control littermates at E13.5 generated comparable numbers of neurospheres, the size of neurospheres from cKO was notably smaller than those from controls (Fig 4G–4I). Subsequently, we dissociated and passaged the obtained neurospheres, and consistently, the size of neurospheres in *Ybx1* cKO was significantly reduced, while the number remained unchanged compared to the control group (Fig 4G, 4J, and 4K). This result aligns with the observed decrease in the mitotic capacity of progenitor cells through in vivo BrdU labeling of *Ybx1* cKO embryos.

Furthermore, we explored another Ybx1 loss-of-function approach by knocking down Ybx1 using in utero electroporation (IUE) in the mouse embryonic cortex. We performed IUE using a plasmid driven by the CAG promoter expressing sh*Ybx1* with GFP reporter at E14.5. Then, we conducted EdU injection and dissection at 24 and 48 h post-electroporation (i.e., E15.5 and E16.5) (Fig 4L). Ybx1 immunofluorescence confirmed efficient knockdown of Ybx1 protein in GFP^+^ cells compared to the control group (Figs 4M and S4G). We then performed immunofluorescence analysis of EdU and Ki67. The results showed a significant reduction in the percentage of EdU^+^GFP^+^/GFP^+^ compared to the control group, indicating decreased proliferation of neural progenitor cells due to Ybx1 knockdown (Fig 4N and 4O). Additionally, we observed a reduced percentage of GFP^+^ cells that were EdU^+^ but Ki67^-^, suggesting fewer cells had exited the cell cycle (Fig 4N and 4P). Furthermore, the percentages of Pax6^+^GFP^+^ representing radial glial cells (RGCs) and Tbr2^+^GFP^+^ representing intermediate progenitor cells (IPCs) in all GFP^+^ cells were also reduced in the sh*Ybx1* cortex (S4H–S4J Fig). These knockdown results were consistent with cKO analysis, further confirming that Ybx1 loss-of-function in the mouse cortex impairs the proliferation and differentiation of neural progenitor cells.

Taken together, these results indicate that Ybx1 plays an important role in regulating the proliferation and differentiation of cortical progenitor cells, and its loss impairs their self-renewal capacity and neurogenesis.

### Ybx1 controls the stability of its m^5^C-modified target transcripts in neural progenitor cells

To elucidate the mechanisms by which Ybx1 regulates NSC proliferation and differentiation, we initially tried to ascertain the subcellular localization of Ybx1 in neural stem cells. Immunostaining of Ybx1 in cultured E13.5 embryonic cortical progenitor cells, marked by Pax6 and Ki67, revealed that Ybx1 protein was predominantly located in the cytoplasm, suggesting a major cytoplasmic function for Ybx1 (S5A and S5B Fig). So, we focused on identifying and elucidating its target mRNAs which Ybx1 regulates in post-transcriptional level.

**Fig 5:**
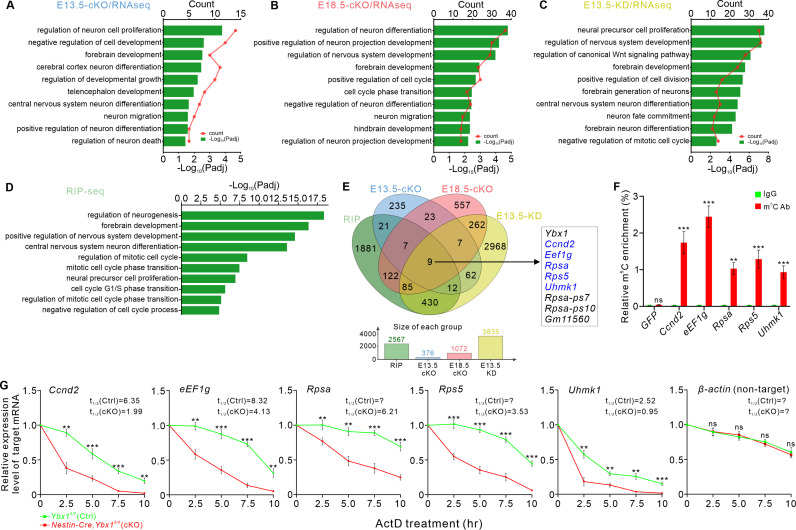
Ybx1 controls the stability of its m^5^C-modified target transcripts in neural progenitor cells. **(A-C)** Gene Ontology (GO) analysis of transcripts with altered expression levels in E13.5 *Ybx1* cKO cortex **(A)**, E18.5 *Ybx1* cKO cortex **(B)**, and E13.5 *Ybx1* KD cortical progenitors **(C)**. GO terms in biological processes were shown. **(D)** GO analysis of Ybx1 target transcripts identified by anti-Ybx1 RIP-seq in the E14.5 mouse cortex. **(E)** Venn diagram showing the overlap of target mRNAs identified by three cKO or KD RNA-seq and anti-Ybx1 RIP-seq. GO terms in biological processes were shown. **(F)** Validation of m^5^C modification on Ybx1 target mRNAs by anti-m^5^C immunoprecipitation combined with RT-qPCR. In vitro transcribed GFP mRNA which has no m^5^C modification was used as a negative control. Data represent mean ± SD (*n* = 3 replicates): ***p = 5.99E−04 for *Ccnd2*; ***p = 1.37E−04 for *eEF1g*; **p = 0.0045 for *Rpsa*; ***p = 8.89E−04 for *Rps5*; ***p = 8.06E−04 for *Uhmk1*; not significant (ns) for *GFP*; by unpaired Student *t* test. **(G)** Ybx1 target transcripts exhibit accelerated degradation in the *Ybx1* cKO cortex. Radial glial cells (RGCs) dissected from E14.5 *Ybx1* cKO and control embryos were cultured, treated with actinomycin D (ActD), and collected at different time points. Ybx1 target mRNA levels were measured by RT-qPCR while *β-actin* mRNA was used a non-target control. Data represent mean ± SEM (*n* = 3 replicates): for *Ccnd2*, **p = 0.0017 (2.5 h), ***p = 7.50E−04 (5 h), ***p = 2.32E−04 (7.5 h), **p = 0.0018 (10 h); for *eEF1g*, **p = 0.0014 (2.5 h), ***p = 3.98E−04 (5 h), ***p = 3.10E−04 (7.5 h), **p = 0.0022 (10 h); for *Rpsa*, **p = 0.0096 (2.5 h), **p = 0.0012 (5 h), ***p = 4.09E−04 (7.5 h), ***p = 5.08E−04 (10 h); for *Rps5*, ***p = 5.67E−04 (2.5 h), ***p = 1.33E−04 (5 h), ***p = 1.65E−04 (7.5 h), ***p = 6.41E−04 (10 h); for *Uhmk1*, **p = 0.0013 (2.5 h), **p = 0.0025 (5 h), **p = 0.0011 (7.5 h), ***p = 7.91E−04 (10 h); for *β-actin*, ns, not significant; by unpaired Student *t* test. The half-lives for each mRNA in Ctrl and cKO were calculated (? indicates that the half-lives cannot be calculated). The data underlying all the graphs shown in the figure are included in S1 Data.

We first performed transcriptome analysis of cortical tissues from both *Ybx1* cKO and control littermates at E13.5 (E13.5-cKO) and E18.5 (E18.5-cKO). Additionally, in order to rule out the possible compensatory effects brought by ablation of *Ybx1* in embryos, we also conducted transcriptome analysis of E13.5 embryonic neural stem cells cultured in vitro after *Ybx1* knockdown (KD) using lentiviral shRNA (E13.5-KD). RNA sequencing (RNA-seq) revealed 161 upregulated genes and 215 downregulated genes in E13.5-cKO compared to the control group (S1 Table), 645 upregulated genes and 427 downregulated genes in E18.5-cKO (S2 Table), and 1886 upregulated genes and 1949 downregulated genes in E13.5-KD (S3 Table). Gene Ontology (GO) analysis of the differentially expressed genes (DEGs) across the three cKO/KD-RNAseq experiments showed significant enrichment in biological processes such as regulation of nervous system development, neural precursor cell proliferation, cerebral cortex neuron differentiation, and forebrain development (Fig 5A–5C). This aligns with the observed impact of *Ybx1* ablation on cortical development. Subsequently, we performed RNA immunoprecipitation sequencing (RIP-seq) using Ybx1 antibody to fish target transcripts interacting with Ybx1 in the mouse cortex at E13.5, identifying a total of 2,567 transcripts (S4 Table). Functional annotation of Ybx1 targets revealed enrichment in cellular components such as spindle, mitotic spindle, and cyclin-dependent protein kinase holoenzyme complex; molecular functions including kinase regulator activity and mRNA binding; and biological process such as regulation of neurogenesis, neural precursor cell proliferation, and mitotic cell cycle phase transition (Fig 5D).

Subsequently, we juxtaposed the anti-Ybx1 RIP-seq data with the three cKO/KD-RNAseq datasets to pinpoint mRNA targets which are directly bound and regulated by Ybx1 in cortical development. By integrating RIP-seq results with DEGs from E13.5-cKO, E18.5*-*cKO, and E13.5-KD RNA-seq, we identified 49, 223, and 536 candidate targets, respectively. GO analysis of these candidate targets showed significant enrichment in biological processes related to neurogenesis, mitosis, and cell cycle regulation (S5C–S5E Fig). These GO terms are also in line with the phenotype of impaired proliferation and differentiation of NSCs after ablation of *Ybx1*. By overlapping the DEGs in E13.5-cKO, E13.5-KD, and E18.5-cKO RNAseq with anti-Ybx1 RIP-seq, we obtained 9 target mRNAs (including *Ybx1* itself, a pseudogene of *Ybx1*, and two pseudogenes of *Rpsa*) that were downregulated consistently in different stages and conditions for Ybx1 loss of function (Fig 5E). We focused on the five target mRNAs (*Ccnd2*, *eEF1g*, *Rpsa*, *Rps5*, and *Uhmk1*) in the following analysis.

We first verified downregulation of these targets in Ybx1 loss of function. When *Ybx1* was knocked down in neural stem cells cultured in vitro, the mRNA levels of these five targets were significantly reduced (S6A Fig). Further in vivo analysis of the cortices at E13.5, E15.5, and E17.5 was conducted by RT-qPCR on RNAs extracted from these tissues. The results confirmed decreases in the mRNA levels of *Ccnd2*, *eEF1g*, *Rpsa*, *Rps5*, and *Uhmk1* in *Ybx1* cKO cortices compared to the controls (S6B Fig). These data verified that Ybx1 regulates the target mRNA levels.

The m^5^C modification of these mRNAs was validated by anti-m^5^C pulldown experiment (Fig 5F). Next, we further investigated whether Ybx1 regulation of its target mRNAs depends on m^5^C modification. Previous studies have shown that the only two known m⁵C RNA methyltransferases, Nsun2 and Nsun6, are closely related to mRNA methylation [30–34]. Knockout experiments targeting *Nsun2* and/or *Nsun6* have demonstrated that Nsun2 and Nsun6 act on the m⁵C sites of different subsets of mRNA and together account for nearly all m⁵C modifications in mRNA [33]. We first knocked down *Nsun2* and *Nsun6* in cultured neural stem cells (S6C Fig) to eliminate m^5^C modification. In these Nsun2/6- and thus m^5^C-deficient cells, we continued to perform *Ybx1* knockdown. The results demonstrated that, in the absence of m^5^C modification, the presence or absence of Ybx1 could not affect the target transcript levels anymore (S6D Fig). These data suggest that Ybx1 regulates the target mRNA levels in an m^5^C-dependent manner. As Ybx1 can promote the stability of its m^5^C-modified targets, we continued to test the stability of those target mRNAs in Ybx1 loss of function conditions. RNA stability assays showed an accelerated degradation of these mRNAs in NSCs from *Ybx1* cKO compared with control cells (Fig 5G). Taken together, all these results suggest that Ybx1 normally can enhance the stability of its m^5^C-modified target mRNAs.

### Ybx1 target mRNAs regulate the proliferation and differentiation of cortical progenitor cells

**Fig 6:**
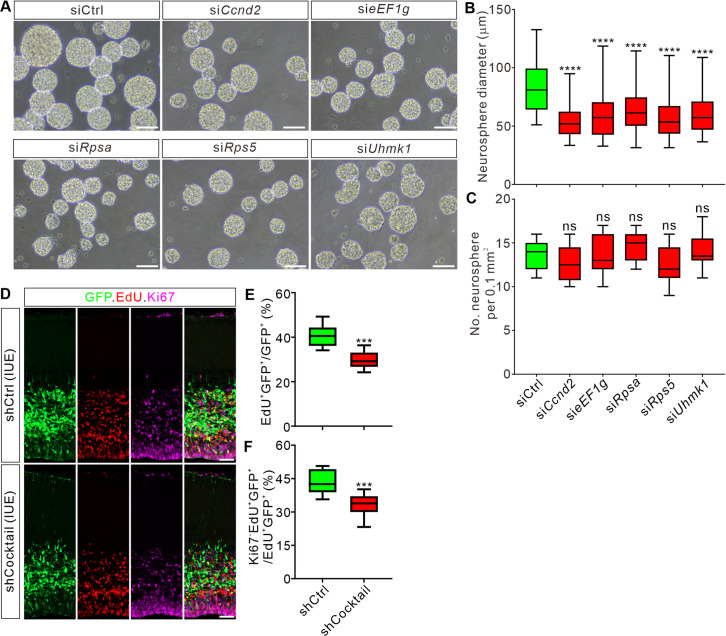
Knockdown of Ybx1 target mRNAs impairs the proliferation and differentiation of cortical progenitor cells. **(A)** Representative images of neurospheres formed after siRNA-mediated knockdown of *Ccnd2*, *eEF1g*, *Rpsa*, *Rps5*, or *Uhmk1* in cultured E13.5 neural stem cells (NSCs). Scale bar, 50 μm. **(B and C)** Quantification of the sizes **(B)** and numbers **(C)** of the neurospheres after siRNA-mediated knockdown of Ybx1 target mRNAs show in **(A)**. At least 3 replicates were performed. All statistical data are presented as box and whisker plots: in **B**, si*Ccnd2* (*n* = 55 neurospheres) vs. siCtrl (*n* = 51 neurospheres), ****p = 5.87E−06; si*eEF1g* (*n* = 52 neurospheres) vs. siCtrl, ****p = 5.46E−07; si*Rpsa* (*n* = 57 neurospheres) vs. siCtrl, ****p = 1.57E−07; si*Rps5* (*n* = 49 neurospheres) vs. siCtrl, ****p = 6.12E−07; si*Uhmk1* (*n* = 45 neurospheres) vs. siCtrl, ****p = 9.87E−08; by one-way ANOVA followed by Tukey’s multiple comparison test; ns, not significant. **(D)** Immunostaining for GFP, EdU, and Ki67 on coronal sections of E16.5 mouse cortex after knockdown of Ybx1 targets in the cortex using IUE of a cocktail shRNA against all targets. Scale bar, 50 μm. **(E and F)** Quantification of percentage of EdU^+^GFP^+^/GFP^+^ **(E)** and Ki67^-^EdU^+^/EdU^+^GFP^+^ **(F)** shown in **(D)**. At least 3 embryos were analyzed for each condition. Data are presented as box and whisker plots: in **E**, shCtrl (*n* = 23 confocal fields) vs. shCocktail (*n* = 27 confocal fields), ***p = 2.10E−04; in **F**, shCtrl (*n* = 23 confocal fields) vs. shCocktail (*n* = 27 confocal fields), ***p = 6.86E−04; by unpaired Student *t* test **(E, F)**. The data underlying all the graphs shown in the figure are included in S1 Data.

Next, we investigated whether the target mRNAs *Ccnd2*, *eEF1g*, *Rpsa*, *Rps5*, and *Uhmk1* could mediate the effects of Ybx1 on proliferation and differentiation of cortical progenitor cells. We generated siRNAs targeting these transcripts and validated their knockdown efficiency (S7A–S7E Fig). Following this, we assessed the influence of target mRNA knockdown on neurosphere formation capacity using these siRNAs. The results showed that knockdown of *Ccnd2*, *eEF1g*, *Rpsa*, *Rps5*, or *Uhmk1* resulted in smaller neurospheres compared to the control siRNA (Fig 6A and 6B), while the numbers remained unchanged (Fig 6A and 6C). Neurosphere assay results suggest that knockdown of Ybx1 targets can inhibit the self-renewal of neural stem cells, recapitulating the neurosphere phenotypes of *Ybx1* cKO.

Furthermore, we explored in vivo functions of Ybx1 target transcripts using in utero electroporation (IUE) experiments in the embryonic cortex. Individual knockdown of *Ccnd2*, *eEF1g*, *Rpsa*, *Rps5*, or *Uhmk1* using specific shRNAs impaired the proliferation and differentiation of neural progenitor cells (except that knockdown of *Rps5* caused a slight but insignificant decrease in differentiation) (S7F and S7G Fig). Interestingly, knocking down all these target mRNAs together with a shRNA cocktail caused more robust reduction in the proliferation and differentiation capacity of neural progenitor cells compared to each individual shRNA (Fig 6D–6F). Together, these results suggest that knockdown of Ybx1 targets in vivo can significantly affect the proliferation and differentiation capabilities of neural stem cells.

### Overexpression of Ybx1 targets rescues the cortical development defects caused by *Ybx1* ablation

**Fig 7:**
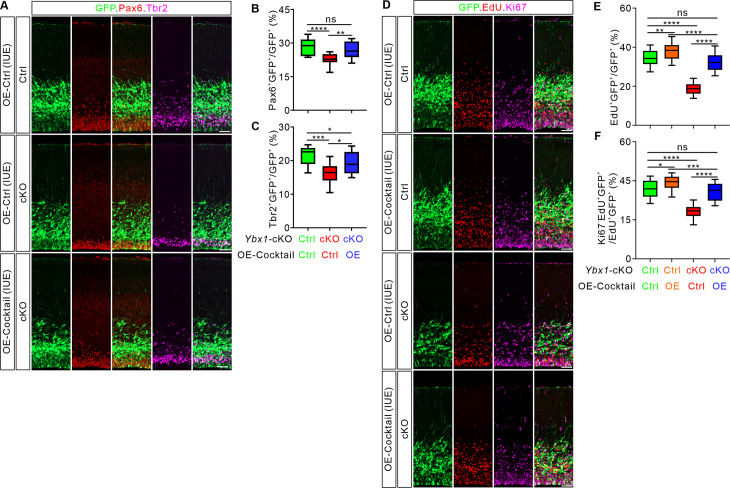
Overexpression of Ybx1 targets rescues the cortical development defects caused by *Ybx1* ablation. **(A)** Overexpression of Ybx1 targets (OE-Cocktail) in *Ybx1* cKO mouse cortex using IUE, followed by immunostaining for GFP, Pax6, and Tbr2 on coronal sections at E16.5. Representative cortical regions were shown. Scale bar, 50 μm. **(B and C)** Quantification of percentage of Pax6^+^GFP^+^/GFP^+^ **(B)** and Tbr2^+^GFP^+^/GFP^+^ **(C)** shown in **(A)**. Data are presented as box and whisker plots. In **B**, “Ctrl + OE-Ctrl” (*n* = 20 confocal fields) vs. “cKO + OE-Ctrl” (*n* = 18 confocal fields), ****p = 4.26E−05; “cKO + OE-Ctrl” vs. “cKO + OE-Cocktail” (*n* = 21 confocal fields), **p = 0.0040; “Ctrl + OE-Ctrl” vs. “cKO + OE-Cocktail”, *p* = 0.10. In **C**, “Ctrl + OE-Ctrl” (*n* = 20 confocal fields) vs. “cKO + OE-Ctrl” (*n* = 18 confocal fields), ***p = 5.69E-04; “cKO + OE-Ctrl” vs. “cKO + OE-Cocktail” (*n* = 21 confocal fields), *p = 0.043; “Ctrl + OE-Ctrl” vs. “cKO + OE-Cocktail”, *p = 0.015. **(D)** Overexpression of Ybx1 targets (OE-Cocktail) in control and *Ybx1* cKO mouse cortex using IUE, followed by immunostaining for GFP, EdU, and Ki67 on coronal sections at E16.5. Representative cortical regions were shown. Scale bar, 50 μm. **(E and F)** Quantification of percentage of EdU^+^GFP^+^/GFP^+^ **(E)** and Ki67^-^EdU^+^/EdU^+^GFP^+^ **(F)** shown in **(D)**. Data are presented as box and whisker plots. In **E**, “Ctrl + OE-Ctrl” (*n* = 27 confocal fields) vs. “Ctrl + OE-Cocktail” (*n* = 25 confocal fields), **p = 0.0099; “Ctrl + OE-Ctrl” vs. “cKO + OE-Ctrl” (*n* = 24 confocal fields), ****p = 3.08E−10; “Ctrl + OE-Ctrl” vs. “cKO + OE-Cocktail” (*n* = 26 confocal fields), *p* = 0.19. “Ctrl + OE-Cocktail” vs. “cKO + OE-Cocktail”, ****p = 8.25E−6. “cKO + OE-Ctrl” vs. “cKO + OE-Cocktail”, ****p = 3.08E−10. In **F**, “Ctrl + OE-Ctrl” (*n* = 26 confocal fields) vs. “Ctrl + OE-Cocktail” (*n* = 28 confocal fields), *p = 0.015; “Ctrl + OE-Ctrl” (*n* = 26 confocal fields) vs. “cKO + OE-Ctrl” (*n* = 24 confocal fields), ****p = 3.08E−10; “Ctrl + OE-Ctrl” vs. “cKO + OE-Cocktail” (*n* = 24 confocal fields), *p* = 0.51. “Ctrl + OE-Cocktail” vs. “cKO + OE-Cocktail”, ***p = 0.00015. “cKO + OE-Ctrl” vs. “cKO +OE-Cocktail”, ****p = 3.08E−10. At least 3 embryos were analyzed for each genotype or condition. Analyses were performed by one-way ANOVA followed by Tukey’s multiple comparison test. ns, not significant. The data underlying all the graphs shown in the figure are included in S1 Data.

We continued to test whether the target mRNAs *Ccnd2*, *eEF1g*, *Rpsa*, *Rps5*, and *Uhmk1* could mediate the effects of Ybx1 on proliferation and differentiation of cortical progenitor cells. Since knockdown of these targets could recapitulate the phenotypes of *Ybx1* cKO, we proceeded to explore whether overexpression of Ybx1 targets could rescue the defects caused by *Ybx1* cKO. We constructed two plasmids overexpressing the Ybx1 targets *Ccnd2* and *eEF1g*, respectively. The results showed that overexpression of either *Ccnd2* or *eEF1g* by IUE could only partially rescue the impaired proliferation and differentiation of cortical progenitor cells caused by *Ybx1* cKO (S8A–S8D Fig). We continued to simultaneously overexpress the five targets *Ccnd2*, *eEF1g*, *Rpsa*, *Rps5*, and *Uhmk1* all together (OE cocktail) in the mouse cortex using IUE. We observed that in the cortex of *Ybx1* cKO embryos overexpressing the cocktail of targets, the proportion of Pax6^+^GFP^+^ and Tbr2^+^GFP^+^ cells among total GFP^+^ cells were equivalent or close to the values in control embryos (Fig 7A–7C). We further conducted EdU pulse labeling experiments and found that the percentage of EdU^+^GFP^+^ cells among all EdU^+^ cells and the proportion of EdU^+^GFP^+^ cells that are Ki67^-^ were also successfully rescued in the *Ybx1* cKO cortex overexpressing the cocktail of targets by IUE (Fig 7D–7F). Additionally, we performed overexpression of the cocktail of targets in the wild type (WT) background, which promoted the proliferation and differentiation of cortical neural stem cells (Fig 7D–7F). Compared with overexpression of the five target genes in the *Ybx1* cKO cortex, overexpression in WT leads to a higher proportion of EdU^+^GFP^+^ cells among EdU^+^ cells, as well as a higher proportion of Ki67^−^ cells among EdU^+^GFP^+^ cells. We believe this difference might be due to the fact that *Ybx1* knockout not only alters the expression levels of these five core target genes but also induces broader transcriptional changes. The downregulation of other genes might counteract the overexpression effects of these five core target genes.

Together, these results indicate that overexpression of Ybx1 target genes can rescue the defective phenotypes of *Ybx1* cKO cortex in cell proliferation and cell cycle exit.

### Ybx1 regulates the G1 to S phase transition of cortical progenitor cell cycle

**Fig 8:**
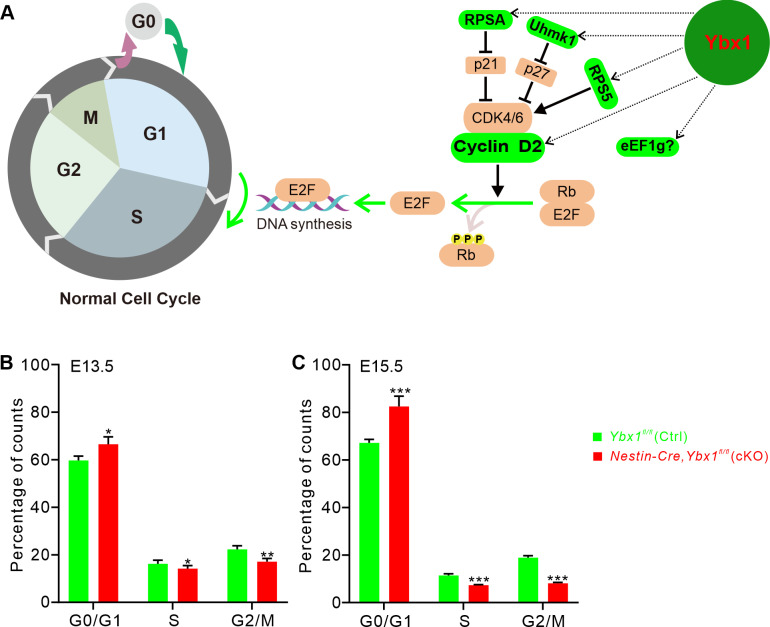
Ybx1 regulates the G1–S phase transition of cortical progenitor cell cycle. **(A)** Schematic drawings showing Ybx1 target mRNAs-encoding proteins are involved in regulating normal cell cycle progression. **(B and C)** Cell cycle flow cytometry analysis determining the percentage of cells in G0/G1, S, and G2/M phases in cortical progenitor cells from E13.5 **(B)** and E15.5 **(C)** *Ybx1* cKO and littermate control embryos. Data are presented as mean ± SEM: *n* = 3 replicates; in **B**, *p = 0.031 for G0/G1 phase, *p = 0.044 for S phase, **p = 0.0012 for G2/M phase; in **C**, ***p = 4.33E−04 for G0/G1 phase, ***p = 9.75E−04 for S phase, ***p = 3.40E−04 for G2/M phase; by unpaired Student *t* test. The data underlying all the graphs shown in the figure are included in S1 Data.

We further investigated the functions of the Ybx1 targets *Ccnd2*, *eEF1g*, *Rpsa*, *Rps5*, and *Uhmk1*. Previous studies have shown that Ccnd2 (cyclin D2), a D-type cell cycle protein, predominantly binds and activates CDK4 and CDK6 during the G1 phase. This activation leads to the phosphorylation and inactivation of the Rb protein family, releasing and activating E2F to initiate DNA synthesis, thereby enhancing the G1 to S phase transition and promoting cell division [35–38]. There is substantial evidence indicating that some ribosomal proteins, in addition to serving as structural components of the ribosome subunit, also participate in the regulation of cell proliferation and differentiation. Rps5, a ribosomal protein, has been shown to regulate the expression levels of CDK4/6, thereby controlling the G1 to S transition of the cell cycle [39,40]. Rpsa, initially identified as a 67 kD laminin-binding protein, is evolutionarily conserved and later identified as a 37 kD member of the RPS family of ribosomal components. Knockdown of RPSA promotes an increase in p21 expression levels, reinforcing inhibition of CDK4/6 and resulting in G1 phase cell cycle arrest in vitro [41–43]. Uhmk1, also known as KIS, consists of a kinase domain and a putative RNA-binding domain (RBD). Uhmk1 can phosphorylate the cyclin-dependent kinase inhibitor p27, leading to nuclear export and proteasomal degradation of p27 and relieving its inhibitory effect on CDK4/6 [44–47]. Thus, interestingly, four (*Ccnd2*, *Rpsa*, *Rps5*, and *Uhmk1*) out of the five Ybx1 target mRNAs are involved in the G1 to S phase transition in the cell cycle (Fig 8A), consistent with the GO enrichment of biological processes related to the regulation of cell cycle phase transition.

To determine whether Ybx1 regulates the cell cycle process through modulating its targets encoding these cell cycle-related proteins, we performed cell cycle analysis based on flow cytometry using propidium iodide (PI) DNA staining. FACS detection results showed that, compared to the control cortex, the proportion of G1 phase cells increased by 6.8% in E13.5 *Ybx1* cKO embryos (Figs 8B and S9A), and by 15.3% at E15.5 (Figs 8C and S9B), indicating that *Ybx1* deletion causes cell cycle arrest at the G1 phase. Additionally, loss of Ybx1 in E13.5 *Ybx1* cKO embryos resulted in a 2.0% reduction in the S phase cells and a 5.2% reduction in the G2/M phase cells (Figs 8B and S9A). At E15.5, these values decreased by 4.1% and 10.8%, respectively (Figs 8C and S9B). Additionally, we used the dual-labeling method, which allows for more precise measurement of the duration of different cell cycle phases (S9C Fig). We found that in the E14.5 mouse cortex, the total cell cycle duration (*T*_*c*_) and the S phase duration (*T*_*s*_) of control neural stem cells were 16.23 h and 4.29 h, respectively (S9D–S9F Fig). In contrast, *Ybx1* cKO neural stem cells exhibited the total cell cycle duration and the S phase duration of 20.80 h and 4.63 h, respectively (S9D–S9F Fig). This indicates that, compared to control mice, *Ybx1*-deficient neural stem cells spend significantly more time in the cell cycle.

Collectively, these findings suggest Ybx1-deficient cortical progenitor cells exhibit a G1-to-S phase blockade in vivo, delayed cell cycle exit, and an extended cell cycle duration, ultimately resulting in the inhibition of cell cycle progression.

Taken together, Ybx1 facilitates the G1–S transition by stabilizing the expression of its target mRNAs encoding the pivotal regulatory factors, which is essential for the normal progression of the cell cycle in neural stem cells.

## Discussion

In addition to the impaired cortical neurogenesis, the *Nestin-Cre*-mediated *Ybx1* cKO mice also show reduced body weight and perinatal lethality phenotypes. These phenotypes might be due to *Ybx1* knockout in non-cortical brain regions. The causes of postnatal death in knockout mice are relatively complex and can be attributed to various factors. Major fatal events in neonates typically include complications related to delivery, respiration, suckling, and neonatal homeostasis, as well as common abnormalities associated with disruptions in these physiological processes [48,49]. To further evaluate the cause of death, one can examine neuromuscular junctions (NMJ), which are crucial for muscle contraction and respiratory function. NMJ dysfunction can lead to motor deficits, abnormal muscle contractions, and breathing difficulties, which may result in rapid death. Furthermore, we observed that *Ybx1* cKO mice exhibit a prominent phenotype of lateral ventricle enlargement. Ventricular enlargement is a major characteristic of hydrocephalus, one of the oldest known neurological disorders in humans, with archaeological records suggesting symptoms of hydrocephalus as far back as ancient Egypt [50]. The etiology of hydrocephalus-induced ventricular enlargement is complex, but recent studies confirm that dysregulation of neural stem cell fate may be a significant factor [50]. Our study also demonstrates that the loss of Ybx1 significantly impacts the maintenance and differentiation of cortical NSCs, underscoring its importance in normal brain development.

Before its association with m^5^C was identified, Ybx1 had already been extensively studied as a multifunctional DNA- and RNA-binding protein involved in various DNA- and RNA-dependent processes. Although it was thought that Ybx1 could regulate transcription [51], its role in transcriptional regulation has been increasingly challenged due to its nonspecific binding to DNA [52,53]. In this study, we explored the subcellular localization of Ybx1 in neural stem cells, revealing that Ybx1 protein is predominantly expressed in the cytoplasm. This suggests that, at least in neural stem cells, Ybx1 mainly has cytoplasmic functions.

Previous studies indicated that *Ybx1* null mutant mice exhibit embryonic lethality, highlighting the importance of Ybx1 in early embryonic development [26,27]. Another study using global *Ybx1* knockout mice investigated the impact of Ybx1 loss on neural stem cells, concluding that Ybx1 deficiency promotes neural stem cell proliferation [54], which is inconsistent with our results showing that *Ybx1* cKO inhibits neural stem cell proliferation. The main difference between our study and that study is that the latter focused on nuclear Ybx1, whereas our study shows that Ybx1 is predominantly located in the cytoplasm of neural stem cells (S5A and S5B Fig), and its negligible presence in the nucleus suggests that its transcriptional function may be questionable. In this project, we utilized Cre recombinase for targeted deletion of *Ybx1* in neural stem cells, facilitating a thorough and accurate investigation into the effects of Ybx1 on cortical development. Our results demonstrated that *Ybx1* ablation inhibits the proliferation and differentiation of neural stem cells, aligning with the essential role of Ybx1 in maintaining germ line stem cell (GSC) proliferation and differentiation in *Drosophila* [23].

Studies have demonstrated that Ybx1 plays critical roles in multiple aspects of RNA metabolism, including splicing, mRNA stability, and translation [55]. Ybx1 can stabilize mRNAs by binding to their 3′ UTRs, thereby preventing the activation of degradation pathways [56,57]. Moreover, as an RNA-binding protein (RBP), Ybx1’s role extends beyond regulating mRNA stability, with studies indicating its significant influence on mRNA translation. For instance, Ybx1 enhances translation initiation efficiency by binding to the 5′ untranslated regions (UTRs) of target mRNAs and is thought to regulate global translation through these interactions [58]. A study on epidermal cells showed that Ybx1 regulates the translation of a subset of mRNAs encoding relevant cell factors, thereby maintaining the proliferative capacity of epidermal progenitor cells [59].

In addition, we identified the target mRNAs regulated by Ybx1 that contribute to the maintenance of cortical stem cell proliferation and differentiation, including proteins related to cell cycle regulation such as Ccnd2. Recent studies on rodents and primates have highlighted the crucial role of cell cycle regulation, particularly on the G1 phase, in controlling the rate of neuron production and the formation of cellular structural patterns [60,61]. Our current study suggests that Ybx1 directly modulates the G1-to-S phase transition in the cell cycle by stabilizing m^5^C-modified transcripts of crucial cell cycle regulators. As mentioned earlier, four targets—*Ccnd2*, *Rpsa*, *Rps5*, and *Uhmk1*—play roles in regulating the G1-to-S phase transition. As for eEF1g protein, there is limited information available. Interestingly, we noticed that eEF1a, belonging to the same family as eEF1g, has been studied for its impact on tumor cell proliferation by regulating the G1-to-S phase transition [60,61]. Thus, the potential association of eEF1g with the cell cycle and its functional mechanisms deserves further investigation.

Our study offers in vivo validation within a mammalian model, bolstering the emerging concept that m^5^C methylation plays an important role in dictating the fate of neural stem cells. The exact and foreseeable progression timeline of cortical neurogenesis necessitates swift and strictly regulated alterations in gene expression [62]. Our findings suggest that the m^5^C-recognizing protein Ybx1, by regulating mRNA stability, provides a critical mechanism for modulating dynamic gene expression and consequently impacting the cell cycle dynamics of mouse neural stem cells. This provides a mechanistic inspiration for future investigations to persist in probing the correlation between this epitranscriptomic regulation and neurological disorders.

## Materials and methods

### Ethics statement

All experiments involving mice were conducted following the approved animal protocol (SUSTech-JY202202059) by the Laboratory Animal Welfare and Ethics Committee of the Southern University of Science and Technology.

### Animals

For the generation of *Ybx1* conditional knockout (cKO) mice, targeted deletion was performed on exons 3–8 of the mouse *Ybx1* gene, which encompasses a 745 bp coding sequence. Deletion of this region would result in the disruption of protein function. Briefly, sgRNAs were transcribed in vitro, and a donor vector was constructed. Cas9, sgRNAs, and the donor were microinjected into fertilized eggs of C57BL/6J mice. Transplanted fertilized eggs were used to obtain *F*_0_ mice, confirmed positive through PCR and sequencing. Positive F0 mice were bred with C57BL/6J mice to obtain stable F1 mouse models. *Ybx1*^*+/fl*^ mice and corresponding *Cre* mice were used to produce *Ybx1* cKO and littermate controls. Genotyping primers were as follows: the first Ybx1-loxP site, 5′-CCAATAGTGACGAAGGTGGAGTGG-3′ and 5′-AGCTTCCACACTATTGCCTGGG-3′; the second Ybx1-loxP site, 5′-GACTAATGGCTGCTAGTTGTCCG-3′ and 5′-AGGGTTTCTCTGTGTAGCTCTGGC-3′. The *Nestin-Cre* mice used in this study were obtained from Jackson Laboratory with stock number 003771. For timed pregnancy, embryonic day E0.5 was considered when the mating plug was observed at noon.

### In situ hybridization

In situ hybridization (ISH) with DIG-labeled RNA probes was performed on mouse tissue sections following a previously reported protocol [63]. The PCR primers used to clone the template for the mouse *Ybx1* ISH probe were 5′-GGTCCTCCACGCAATTACCA-3′ and 5′-TGTTGGATGACCAAACCGGA-3′. RNA probes were transcribed in vitro using the DIG RNA labeling Kit (SP6/T7) (Roche). Tissue section preparation for ISH followed a similar procedure to immunofluorescence, but the entire process used RNase-free reagents. Briefly, the procedure involved fixing tissue sections with 4% paraformaldehyde (PFA) followed by treatment with Proteinase K. The sections were then acetylated with acetic anhydride. Subsequently, the sections were pre-hybridized at 65 °C for 2 h in hybridization buffer, followed by overnight incubation at 65 °C with fresh hybridization buffer containing the probes. The next day, the sections were washed with 2× SSC and treated with 10 μg/mL RNase A at 37 °C for 8–10 min. After sequential washes with 2× SSC, 0.2× SSC, and PBT buffers, the sections were blocked with 10% heat-inactivated sheep serum at room temperature for 1 h. The sections were then incubated overnight at 4 °C with anti-digoxigenin diluted in 10% sheep serum. On the third day, after washing with PBT and alkaline phosphatase buffer, color reaction was performed using NBT/BCIP diluted in alkaline phosphatase buffer in the dark. The color reaction was stopped with PBS when the target gene signal appeared, and the sections were mounted for observation. Both anti-digoxigenin-AP and NBT/BCIP stock solutions were obtained from Roche. In situ hybridization images were collected using the TissueFAXS microscope (TissueGnostics), with the same settings for each group in the experiment.

### Immunostaining

For mouse brain tissues obtained from embryos, fixation was carried out overnight in 4% paraformaldehyde (PFA) at 4 °C. After washing with PBS, mouse brains were dehydrated in 30% sucrose at 4 °C for one day, embedded in O.C.T. (SAKURA), and frozen-sectioned at a thickness of 12 μm using a Leica CM1950 cryostat. When antigen retrieval was required for certain antibodies, brain sections were treated with sodium citrate buffer (pH 6.0) at 90 °C for 20 min and then cooled to room temperature. For immunostaining, sections were first blocked in blocking buffer (10% donkey serum, 5% BSA, and 0.25% Triton X-100) at room temperature for 1–2 h, followed by overnight incubation with primary antibodies (diluted in blocking buffer) at 4 °C. After washing 3 × 10 min in PBS, secondary antibodies such as Alexa Fluor 488, 568, or 647 (Invitrogen) were added (1:500) and incubated at room temperature for 2 h. Following two PBS washes, sections were mounted using anti-fade mounting medium with DAPI (Beyotime). For cultured neurons, subsequent to two PBS washes, cells were fixed using 4% PFA for 15 min at room temperature, followed by two additional PBS washes and a 30-minute blocking at room temperature in blocking buffer. Subsequent antibody incubation conditions were similar to tissue sections. All images were acquired using Zeiss LSM 800 confocal microscope or TissueFAXS microscope. In the same experiment, each group had the same settings. For tissues requiring fluorescence intensity analysis, ImageJ software was used to measure immunofluorescence intensity, subtracting background intensity. Antibody sources and dilutions were as follows: Ybx1 (Abcam, ab76149, 1:500), Pax6 (BD Biosciences, 561,462, 1:500), Tbr2 (Abcam, ab183991, 1:500), Ctip2 (Abcam, ab18465, 1:500), Tbr1 (Abcam, ab31940, 1:500), Cleaved caspase-3 (Cell Signaling, 9661S, 1:500), BrdU (Abcam, ab6326, 1:500), BrdU (Thermo, B35128, 1:250), Ki67 (Cell Signaling, 12202S, 1:500), GFP (Abcam, ab13970, 1:500).

### TUNEL assay

Apoptosis was detected using the TUNEL Apoptosis Detection Kit (Roche), following the manufacturer’s protocol. In brief, sections were fixed with 4% PFA, followed by blocking at room temperature for 1 h in blocking buffer (5% BSA and 0.25% Triton X-100). The TUNEL reaction mixture was added and incubated at 37 °C for 30 min. Subsequently, samples were stained with DAPI, and imaged using a Zeiss LSM 800 confocal microscope.

### BrdU pulse labeling assay

Pregnant mice were intraperitoneally injected with BrdU dissolved in PBS (100 mg/kg), and embryonic brain tissues were harvested 24 h post-injection. For BrdU detection, antigen retrieval was performed using sodium citrate buffer (pH 6.0). Following thawing, slides were washed in PBS for 5 min at room temperature. Samples were then incubated in 2N HCl at room temperature for 30 min, followed by neutralization in 0.1 M borate buffer for 10 min. After washing with PBS, the samples underwent the same blocking and antibody incubation process as in immunofluorescence staining.

### Neurosphere assay

Cortical tissues from E13.5 embryos were dissected and dissociated using Accutase (Sigma) at 37 °C. Cells were then cultured in NeuroCult medium (STEMCELL Tech) supplemented with EGF (20 ng/mL, Gibco) and bFGF (10 ng/mL, Gibco). Cultures were refreshed every two days with 100 μL of medium, and neurospheres were imaged on day 7. For clonal analysis, primary neurospheres were dissociated into single cells using Accutase for 5 min and suspended in fresh culture medium. After 5 days, newly formed secondary neurospheres were photographed and quantitatively analyzed.

### Cortical culture

Cortical tissues from mouse embryos were isolated using Leibovitz’s L-15 medium, followed by dissociation in HBSS supplemented with trypsin (Sigma) and DNase I (Sigma). Dissociated cortical cells were plated on glass coverslips or culture dishes pre-coated with poly-D-lysine (100 μg/ml, Trevigen) and laminin (3.3 μg/ml, Trevigen). Cultures were maintained in neurobasal medium (Gibco) supplemented with B27 (Thermo Fisher), GlutaMAX supplement (Thermo Fisher), and penicillin–streptomycin (Thermo Fisher). The medium was changed every two days with fresh culture medium.

### Gene knockdown using lentiviral shRNA or siRNA

Lentiviral vectors for gene knockdown were prepared using the pLKO.1-Puro system (Addgene). Detailed procedures for lentivirus preparation are described in previous literature [13,18]. Target sequences for shRNA were as follows:

- sh*Ybx1* #1: 5′‐GGTATCGCCGAAACTTCAATT‐3′
- sh*Ybx1* #2: 5′‐GTATCGCCGAAACTTCAATTA‐3′
- shCtrl: 5′‐GCATAAACCCGCCACTCATCT‐3′

To select positively infected neurons, puromycin (1 μg/mL, Sigma) was added on the second day post-infection and treated for 24 h before sample collection for analysis.

Gene silencing experiments, employing siRNA-mediated knockdown of *Ccnd2*, *eEF1g*, *Rpsa*, *Rps5*, and *Uhmk1*, were performed using GP-transfect-Mate transfection reagent (Genepharma) following the manufacturer’s manual. The target sequences for siRNA were as follows:

- siCtrl: 5′-UUCUCCGAACGUGUCACGUTT-3′
- si*Ybx1* #1: 5′-GUAUCGCCGAAACUUCAAUUATT-3′
- si*Ybx1* #2: 5′-GGUAUCGCCGAAACUUCAAUUTT-3′
- si*Nsun2*: 5′-GCUCACAACACUGAGAACATT-3′
- si*Nsun6*: 5′-GCGAAGGUUUCUCAGAAAUTT-3′
- si*Ccnd2* #1: 5′-CGACUUCAAGUUUGCCAUGUATT-3′
- si*Ccnd2* #2: 5′-CAUUGAGCACAUCCUUCGCAATT-3′
- si*eEF1g* #1: 5′-GAGUGACACUGGCUGAUAUUATT-3′
- si*eEF1g* #2: 5′-GUGGUUCCUUACCUGCAUUAATT-3′
- si*Rpsa* #1: 5′-AGUGACGGUAUCUACAUCAUATT-3′
- si*Rpsa* #2: 5′-CCUGAUCUUUACUUCUACAGATT-3′
- si*Rps5* #1: 5′-GCGCCUUACUAACUCCAUGAUTT-3′
- si*Rps5* #2: 5′-GCCUUACUAACUCCAUGAUGATT-3′
- si*Uhmk1* #1: 5′-UAUCACCUCAGAGACCUUAUCTT-3′
- si*Uhmk1* #2: 5′-GAGUGCGGAGAAUGAGUGUUUTT-3′

Cells were incubated with the transfection solution for 4–6 h before replacing the medium and then samples were collected after 48 h for subsequent analysis.

### RNA sequencing and data analysis

For RNA sequencing, cortices from E13.5 or E18.5 mouse embryos were dissected, and genotyping was conducted to differentiate between *Ybx1* cKO and wild type control littermates. Tissues were lysed using Trizol reagent. Cortical cultures with Ybx1 knockdown were obtained as described earlier, and total RNA was extracted from cultured cortical cells infected with shRNA lentivirus after puromycin selection using Trizol reagent. Subsequently, mRNA purification, cDNA library construction, sequencing, and analysis were conducted. RNA sequencing was performed using the DNBSEQ platform from BGI, following standard procedures. The results of the gene expression profiles were derived from three independent replicate experiments.

### RNA immunoprecipitation and sequencing (RIP-seq)

RIP experiments were conducted using the EZ-Magna RIP RNA-Binding Protein Immunoprecipitation Kit (Millipore), in accordance with a previously established protocol [16]. In brief, 2 × 10^7^ cells were lysed, and the lysates were incubated overnight at 4 °C with Ybx1 antibody (Abcam). Subsequently, immunoprecipitated RNA samples were used for cDNA library construction, sequencing, and analysis. Sequencing results for each group were derived from two independent replicate experiments. All enriched genes were subjected to Gene Ontology (GO) analysis, and gene length bias correction was performed using the GOseq R package. GO terms with corrected *P* values less than 0.05 were considered significantly enriched.

### m^5^C mRNA immunoprecipitation

m^5^C mRNA immunoprecipitation was performed using the GenSeq m^5^C MeRIP Kit (GenSeq), following the manufacturer’s instructions. In summary, cortical RNA was extracted from E14.5 wild type mouse pups. The experiment involved the use of m^5^C antibody and corresponding control IgG (both included in the kit). *GFP* mRNA was in vitro transcribed using the DIG RNA labeling Kit (Roche) and used as a negative control to ensure the absence of m^5^C modifications. The immunoprecipitated RNA was then subjected to cDNA synthesis and quantitative real-time PCR based on SYBR Green.

### RT-qPCR analysis

Total RNA from cortical tissues or cultured cortical cells was extracted using Trizol Reagent (Life). cDNA was synthesized with the PrimeScript RT Master Mix (Takara), and real-time quantitative PCR (qPCR) was performed using the ChamQ Universal SYBR qPCR Master Mix (Vazyme) on a BioRad qPCR CFX 96 instrument. qPCR results were quantified using the 2^−ΔΔCt^ method, calculating the difference in threshold cycle (Ct) values between the target gene and an internal control. The primer sequences used for qPCR were as follows: *Ybx1*: 5′‐GAGAGGATGGCAATGAAGAGG‐3′ and 5′‐TTGTGGTTTAGGGTTCTCTGG‐3′; *Ccnd2*: 5′‐GAGTGGGAACTGGTAGTGTTG‐3′ and 5′‐CGCACAGAGCGATGAAGGT‐3′; *eEF1g*: 5′‐ACCGCACCCCTGAATTTCTC‐3′ and 5′‐CTGGCGTACTTCCTCGCAG‐3′; *Rpsa*: 5′‐TGCGGGAACCCACTTAGGT‐3′ and 5′‐AGGATTCTCGATGGCAACAATAG‐3′; *Rps5*: 5′‐TGGCAGAGACCCCTGACAT‐3′ and 5′‐GGGCAGGTACTTGGCATACT‐3′; *Uhmk1*: 5′‐CTCTCCCAATGTGCCATCAC‐3′ and 5′‐TTCGTGGTTTGAGGTCTGCAT‐3′; *β-actin*: 5′‐GCGAGCACAGCTTCTTTGC‐3′ and 5′‐TCGTCATCCATGGCGAACT‐3′; *GAPDH*: 5′‐CAAGGAGTAAGAAACCCTGGAC‐3′ and 5′‐GGATGGAAATTGTGAGGGAG‐3′.

### RNA stability assay

Cortical cells, isolated from mouse brain tissue, were plated in equal numbers per well and allowed to adhere. Cultured cells were treated with actinomycin D (ActD, ApexBio Technology) at a concentration of 5 μg/ml. Cells were collected at 0, 2.5, 5, 7.5, and 10 h, and RNA was isolated for qPCR analysis.

### In utero electroporation and EdU assay

Plasmid transfection via intraventricular injection followed by in utero electroporation was performed as previously described [64]. In brief, the plasmids were diluted in Fast Green (Sigma–Aldrich) at a ratio of 1:20 to achieve a final concentration of 1 μg/μl. Pregnant E14.5 mice were anesthetized, and the uterine horns were exposed. A pulled glass micropipette was used to inject 1 μl of DNA solution into the lateral ventricle of the embryos. Tweezers (5 mm platinum, BTX, Harvard Apparatus) were positioned at the injection site, and the brain was electroporated using the BTX-EC830 electroporator (BTX, Harvard Apparatus) with five pulses at 40 V, 50 ms duration, and 950 ms intervals to introduce DNA into the cortex. Embryos were quickly returned to the abdominal cavity to minimize temperature loss. The abdominal wall and skin were sutured, allowing normal fetal development. To assess cell cycle exit, pregnant mice received an injection of EdU at a dose of 25 mg/kg 24 h after electroporation. Brains were collected and processed 24 h after injection, and EdU incorporation was detected using the Click-iT EdU Alexa Fluor Imaging Kit (Invitrogen).

### Flow cytometry

For cell cycle analysis, neural stem cells were obtained from the mouse cortex and fixed overnight in 70% ethanol at −20 °C. Cells were washed with cold PBS, and the pellet was subsequently resuspended in FxCycle PI/RNase staining solution (Thermo Fisher Scientific), followed by a 30 min incubation at 37 °C. The cell suspension was filtered through a sterile cell strainer (40 μm) for analysis using the BD FACSAria flow cytometer, and the cell cycle distribution of neural stem cells was analyzed using FlowJo software.

### Calculation of cell cycle kinetics

For the analysis of cell cycle dynamics in the embryonic cortex, a BrdU pulse (100 mg/kg body weight) was administered intraperitoneally to the pregnant dam on the day of the experiment. After 1.5 h, bromodeoxyuridine (EdU, 25 mg/kg body weight) was injected, and the embryos were sacrificed 30 min later. Detection of BrdU and EdU in the double-labeling experiments was performed according to the method described above.

Cell cycle duration was calculated using the following paradigm [65]. During the interval between BrdU and EdU (*T*_*i*_ = 1.5 h), cells in the initial BrdU-labeled cohort that exit S phase are designated as the leaving fraction (L cells), which will be labeled with BrdU but not EdU. Cells labeled with both BrdU and EdU are designated as S cells. The ratio of the duration of any phase of the cell cycle to another phase is equal to the ratio of the number of cells in that phase to the number of cells in the other phase [66]. Therefore, the ratio between the number of L cells and S cells is equal to the ratio of *T*_*i*_ (1.5 h) to *T*_*s*_ [67]. S phase duration (*T*_*s*_) can be calculated using the following formula: *T*_*S*_(1.5) = (S cells)/(L cells). The total cell cycle duration (*T*_*c*_) can be estimated using the following formula: *T*_*C*_/*T*_*S*_ = (P cells)/(S cells), where P cells represent the total number of proliferating cells in the sampling area.

### Quantification and statistical analysis

Unless otherwise specified, all values are presented as the mean ± SEM. Statistical analyses were conducted utilizing GraphPad Prism version 8.0. For the comparison of means between two groups, unpaired Student *t* test was employed based on the experimental design. When comparing means among more than two groups, one-way analysis of variance (ANOVA) was conducted, followed by Tukey’s multiple comparison test. A *p* value less than 0.05 was considered statistically significant: *p < 0.05, **p < 0.01, ***p < 0.001, ****p < 0.0001.

## Supporting information

S1 Fig Expression of Ybx1 in VZ and SVZ, and construction and characterization of *Ybx1* conditional knockout (cKO) mice (Related to Fig 1). **(A)** Adjacent coronal brain sections from wild type (WT) mouse embryos at E13.5 and E15.5 were immunostained with antibodies against Ybx1, and Pax6 and Tbr2, respectively. Scale bars, 100 μm. **(B)** The exons 3 to 8 of the *Ybx1* gene were deleted after Nestin-Cre-mediated recombination, resulting in the generation of *Ybx1* conditional knockout (cKO) mice. **(C)** Representative images of P0 sagittal brain sections stained with DAPI. The asterisk indicates the enlarged ventricle in the cKO brain. Scale bar, 500 μm.
(TIF)

S2 Fig Cell death shows no difference among different genotypes (Related to Fig 2). **(A)** Representative images of TUNEL staining in P0 cortex. Scale bar, 100 μm. **(B)** Quantification of TUNEL signals in P0 cortex is presented as a box and whisker plot. **(C)** Representative images of cleaved caspase-3 (CC3) staining in E13.5 cortex. Scale bar, 500 μm. (**D**) Quantification of CC3 signals in E13.5 cortex is presented as a box and whisker plot. **(E)** Representative images of CC3 staining in E15.5 cortex. Scale bar, 500 μm. (**F**) Quantification of CC3^+^ signals in E15.5 cortex is presented as a box and whisker plot. At least 3 mice were analyzed for each genotype. All analyses were performed by one-way ANOVA followed by Tukey’s multiple comparison test. ns, not significant. The data underlying all the graphs shown in the figure are included in S1 Data.
(TIF)

S3 Fig Ybx1 is successfully ablated from cortex starting from E13.5 (Related to Fig 3). **(A)** Coronal brain sections at E11.5, E13.5, E15.5, and E17.5 were stained with antibodies against Ybx1 and Pax6. Representative cortical regions were shown. Scale bars, 100 μm. **(B)** Coronal brain sections at E11.5 were stained with antibodies against Ybx1 and Pax6. Scale bar, 500 μm. **(C and D)** Quantification of Pax6^+^ cell numbers **(C)** and whole cortical thickness **(D)** shown in **(B)**. All statistical data are presented as box and whisker plots. ns, not significant. **(E–M)** Quantification of Pax6^+^ **(E)** and Tbr2^+^ **(F)** cell numbers, and cortical layer thicknesses **(G to M)** at E17.5. All statistical data are presented as box and whisker plots. For Pax6, Ctrl (*n* = 15 confocal fields) versus cKO (*n* = 14 confocal fields), ***p = 5.42E−04; Het (*n* = 15 confocal fields) versus cKO, **p = 0.0033. For Tbr2, Ctrl (*n* = 15 confocal fields) versus cKO (*n* = 14 confocal fields), ***p = 9.97E−04; Het (*n* = 15 confocal fields) versus cKO, ***p = 2.77E−04. For VZ thickness, Ctrl (*n* = 15 confocal fields) versus cKO (*n* = 14 confocal fields), ***p = 8.39E−04; Het (*n* = 15 confocal fields) versus cKO, **p = 0.0031. For SVZ thickness, Ctrl (*n* = 15 confocal fields) versus cKO (*n* = 14 confocal fields), ***p = 8.84E−04; Het (*n* = 15 confocal fields) versus cKO, **p = 0.0010. For IZ thickness, Ctrl (*n* = 18 confocal fields) versus cKO (*n* = 15 confocal fields), ***p = 2.46E−04; Het (*n* = 17 confocal fields) versus cKO, **p = 0.0036. For VI thickness, Ctrl (*n* = 18 confocal fields) versus cKO (*n* = 15 confocal fields), **p = 0.0016; Het (*n* = 17 confocal fields) versus cKO, **p = 0.0070. For V thickness, Ctrl (*n* = 18 confocal fields) versus cKO (*n* = 15 confocal fields), ***p = 3.89E−04; Het (*n* = 17 confocal fields) versus cKO, ***p = 2.82E−04. For I–IV thickness, Ctrl (*n* = 18 confocal fields) versus cKO (*n* = 15 confocal fields), ***p = 8.58E−04; Het (*n* = 17 confocal fields) versus cKO, **p = 0.0012. For whole cortical thickness, Ctrl (*n* = 18 confocal fields) versus cKO (*n* = 15 confocal fields), ****p = 4.07E−05; Het (*n* = 17 confocal fields) versus cKO, ***p = 2.03E−04. **(N)** A stacked bar chart summarizing the data in **(K–O)** shows the distribution of each layer in E17.5 cortex. At least 3 embryos were analyzed for each genotype. All analyses were performed by one-way ANOVA followed by Tukey’s multiple comparison test. ns, not significant. The data underlying all the graphs shown in the figure are included in S1 Data.
(TIF)

S4 Fig Ybx1 regulates proliferation and differentiation of cortical progenitor cells at E15.5 and E17.5 (Related to Fig 4). **(A)** Coronal brain sections at E15.5 were stained with antibodies recognizing BrdU and Ki67. Pregnant mothers received a BrdU pulse 24 h before embryo collection. Representative cortical regions were shown. Scale bar, 100 μm. **(B and C)** Quantification of BrdU^+^ cell numbers **(B)** and the percentage of cells exiting the cell cycle **(C)** at E15.5 shown in **(A)**. All statistical data are presented as box and whisker plots. For BrdU^+^ cell numbers, Ctrl (*n* = 26 confocal fields) versus cKO (*n* = 23 confocal fields), ****p = 6.15E−05; Het (*n* = 21 confocal fields) versus cKO, ***p = 7.86E−04. For Ki67^−^BrdU^+^/BrdU^+^, Ctrl (*n* = 26 confocal fields) versus cKO (*n* = 23 confocal fields), ***p = 2.36E−04; Het (*n* = 21 confocal fields) versus cKO, **p = 0.0017. **(D)** Coronal brain sections at E17.5 were stained with antibodies recognizing BrdU and Ki67. Pregnant mothers received a BrdU pulse 24 h before embryo collection. Representative cortical regions were shown. Scale bar, 50 μm. **(E and F)** Quantification of BrdU^+^ cell numbers **(E)** and the percentage of cells exiting the cell cycle **(F)** at E17.5 shown in **(D)**. All statistical data are presented as box and whisker plots. For BrdU^+^ cell numbers, Ctrl (*n* = 20 confocal fields) versus cKO (*n* = 19 confocal fields), ***p = 6.01E−04; Het (*n* = 19 confocal fields) versus cKO, **p = 0.0025. For Ki67^−^BrdU^+^/BrdU^+^, Ctrl (*n* = 20 confocal fields) versus cKO (*n* = 19 confocal fields), ***p = 2.55E−04; Het (*n* = 19 confocal fields) versus cKO, **p = 0.0015. **(G)** Quantification of Ybx1 immunofluorescence intensity in the cortex of sh*Ybx1* embryos and controls. Data are presented as box and whisker plots: shCtrl (*n* = 29 confocal fields) versus sh*Ybx1* (*n* = 27 confocal fields), ****p = 7.81E−09. **(H)** Immunostaining for GFP, Pax6, and Tbr2 on coronal sections of E16.5 mouse cortex after knockdown of Ybx1 using IUE. Representative cortical regions were shown. Scale bar, 50 μm. **(I and J)** Quantification of percentage of Pax6^+^GFP^+^/GFP^+^ **(I)** and Tbr2^+^GFP^+^/GFP^+^ **(J)** shown in **(H)**. Data are presented as box and whisker plots: in **I**, shCtrl (*n* = 17 confocal fields) versus sh*Ybx1* (*n* = 19 confocal fields), ***p = 5.67E−04; in **J**, shCtrl (*n* = 17 confocal fields) versus sh*Ybx1* (*n* = 19 confocal fields), **p = 0.0046. At least 3 pups or embryos were analyzed for each genotype or condition. Analyses were performed by one-way ANOVA followed by Tukey’s multiple comparison test **(B, C; E, F)**, or by unpaired Student *t* test **(G, I, J)**. ns, not significant. The data underlying all the graphs shown in the figure are included in S1 Data.
(TIF)

S5 Fig Ybx1 protein is predominantly located in the cytoplasm of neural progenitor cells (Related to Fig 5). **(A and B)** Ybx1 immunostaining in cultured neural progenitor cells marked by Pax6 and Ki67 showed predominant signals in cytoplasm. The signals were absent in the cells from the *Ybx1* cKO embryos, suggesting the specificity of Ybx1 immunofluorescence. Scale bars, 5 μm. **(C–E)** Venn diagrams showed the overlap of mRNAs identified by anti-Ybx1 RIP-seq with RNA-seq of *Ybx1* cKO at E13.5 **(C)** and E18.5 **(D)**, and *Ybx1* KD at E13.5 **(E)**. GO terms in biological processes were shown for these overlapped mRNAs. The data underlying all the graphs shown in the figure are included in S1 Data.
(TIF)

S6 Fig Ybx1 regulation of its target mRNAs is m^5^C-depdendent (Related to Fig 5). **(A)** RT-qPCR confirmed the decreased expression levels of Ybx1 target mRNAs in neural stem cells with Ybx1 knockdown using siRNA. Data are presented as mean ± SEM (*n* = 3 replicates): for *Ccnd2*, ***p = 0.00035; for *eEF1g*, ****p = 3.67E−05; for *Rpsa*, ***p = 0.00043; for *Rps5*, ***p = 0.00054; for *Uhmk1*, **p = 0.0031; by unpaired Student *t* test. **(B)** RT-qPCR confirmed the expression decreases of Ybx1 target mRNAs in the *Ybx1* cKO cortices at E13.5, E15.5, and E17.5. Data are presented as mean ± SEM (*n* = 3 replicates): for *Ccnd2*, **p = 0.0063 (E13.5), ***p = 0.00032 (E15.5), ***p = 0.00023 (E17.5); for *eEF1g*, ***p = 0.00069 (E13.5), ***p = 0.00026 (E15.5), ***p = 0.00032 (E17.5); for *Rpsa*, **p = 0.0026 (E13.5), ***p = 0.00055 (E15.5), ***p = 0.00016 (E17.5); for *Rps5*, *p = 0.021 (E13.5), ***p = 0.00079 (E15.5), ***p = 0.00071 (E17.5); for *Uhmk1*, **p = 0.0012 (E13.5), ***p = 0.00048 (E15.5), ***p = 0.00019 (E17.5); by unpaired Student *t* test. **(C)** RT-qPCR validating knockdown efficiency of siRNAs against *Nsun2* and *Nsun6* in cultured cortical neural stem cells dissected from E13.5 mouse embryos. Data are presented as mean ± SEM: at least 3 replicates were analyzed for each experiment (*n* = 3); for *Nsun2*, ****p = 2.93E−05; for *Nsun6*, ***p = 0.00043; by unpaired Student *t* test. **(D)** Ybx1 could not regulate its target mRNA levels anymore after knockdown of m^5^C readers. Data are presented as mean ± SEM: at least 3 replicates were analyzed for each experiment (*n* = 3); ns, not significant; by unpaired Student *t* test. The data underlying all the graphs shown in the figure are included in S1 Data.
(TIF)

S7 Fig Characterization of Ybx1 target mRNAs (Related to Fig 6). **(A–E)** RT-qPCR validated the knockdown efficiency of siRNAs targeting Ybx1 target mRNAs. Cortical neural stem cells dissected from E13.5 were cultured, transfected with siRNAs, and then RNA was extracted for RT-qPCR. Data are presented as mean ± SEM; at least 3 sets of replicates were analyzed for each experiment (*n* = 3). In **A**, si*Ccnd2*#1 versus siCtrl, ***p = 0.00096; si*Ccnd2*#2 versus siCtrl, ***p = 0.00099. In **B**, si*eEF1g*#1 versus siCtrl, **p = 0.0057; si*eEF1g*#2 versus siCtrl, **p = 0.0013. In **C**, si*Rpsa*#1 versus siCtrl, **p = 0.0011; si*Rpsa*#2 versus siCtrl, **p = 0.0074. In **D**, si*Rps5*#1 versus siCtrl, **p = 0.0021; si*Rps5*#2 versus siCtrl, ***p = 0.00064. In **E**, si*Uhmk1*#1, **p = 0.0087; si*Uhmk1*#1 versus siCtrl, ***p = 0.00057. All by unpaired Student *t* test. **(F and G)** Quantification of percentage of EdU^+^GFP^+^/GFP^+^ **(F)** and Ki67^-^EdU^+^/EdU^+^GFP^+^ **(G)** after knockdown of Ybx1 targets in the cortex using IUE of individual shRNA against each target. At least 3 embryos were analyzed for each condition. Data are presented as box and whisker plots. In **F**, shCtrl (*n* = 25 confocal fields) versus sh*Ccnd2* (*n* = 23 confocal fields), **p = 0.0031; shCtrl (*n* = 20 confocal fields) versus sh*eEF1g* (*n* = 21 confocal fields), **p = 0.0061; shCtrl (*n* = 27 confocal fields) versus sh*Rpsa* (*n* = 23 confocal fields), **p = 0.0052; shCtrl (*n* = 22 confocal fields) versus sh*Rps5* (*n* = 24 confocal fields), *p = 0.030; shCtrl (*n* = 27 confocal fields) versus sh*Uhmk1* (*n* = 26 confocal fields), **p = 0.0011. In **G**, shCtrl (*n* = 25 confocal fields) versus sh*Ccnd2* (*n* = 23 confocal fields), *p = 0.013; shCtrl (*n* = 20 confocal fields) versus sh*eEF1g* (*n* = 21 confocal fields), *p = 0.034; shCtrl (*n* = 27 confocal fields) versus sh*Rpsa* (*n* = 23 confocal fields), **p = 0.0049; shCtrl (*n* = 22 confocal fields) versus sh*Rps5* (*n* = 24 confocal fields), *p* = 0.12; shCtrl (*n* = 27 confocal fields) versus sh*Uhmk1* (*n* = 26 confocal fields), **p = 0.0031. All by unpaired Student *t* test. The data underlying all the graphs shown in the figure are included in S1 Data.
(TIF)

S8 Fig Overexpression of individual target mRNA can only partially restore the proliferation and differentiation of neural stem cells in the *Ybx1* cKO mice (Related to Fig 7). **(A–D)** Quantification of percentage of EdU^+^GFP^+^/GFP^+^ **(A, C)** and Ki67^-^EdU^+^GFP^+^/EdU^+^GFP^+^ **(B, D)** after overexpression of *Ccnd2* **(A, B)** and *eEF1g* **(C, D)** in the cortex using IUE. Data are presented as box and whisker plots. In **A**, “Ctrl + OE-Ctrl” (*n* = 17 confocal fields) versus “cKO + OE-Ctrl” (*n* = 16 confocal fields), ***p = 1.57E−04; “cKO + OE-Ctrl” versus “cKO + OE-Cocktail” (*n* = 19 confocal fields), **p = 0.0052; “Ctrl + OE-Ctrl” versus “cKO + OE-Cocktail”, **p = 0.0046. In **B**, “Ctrl + OE-Ctrl” (*n* = 17 confocal fields) versus “cKO + OE-Ctrl” (*n* = 16 confocal fields), ****p = 5.67E−06; “cKO + OE-Ctrl” versus “cKO + OE-Cocktail” (*n* = 19 confocal fields), *p = 0.027; “Ctrl + OE-Ctrl” versus “cKO + OE-Cocktail”, ***p = 6.97E−04. In **C**, “Ctrl + OE-Ctrl” (*n* = 14 confocal fields) versus “cKO + OE-Ctrl” (*n* = 15 confocal fields), ***p = 2.64E−04; “cKO + OE-Ctrl” versus “cKO + OE-Cocktail” (*n* = 15 confocal fields), *p = 0.022; “Ctrl + OE-Ctrl” versus “cKO + OE-Cocktail”, **p = 0.0015. In **D**, “Ctrl + OE-Ctrl” (*n* = 14 confocal fields) versus “cKO + OE-Ctrl” (*n* = 15 confocal fields), ****p = 6.69E−05; “cKO + OE-Ctrl” versus “cKO + OE-Cocktail” (*n* = 15 confocal fields), **p = 0.0046; “Ctrl + OE-Ctrl” versus “cKO + OE-Cocktail”, ***P = 2.58E−04. Analyses were performed by one-way ANOVA followed by Tukey’s multiple comparison test. At least 3 embryos were analyzed for each genotype or condition. The data underlying all the graphs shown in the figure are included in S1 Data.
(TIF)

S9 Fig The cell cycle kinetics of cortical progenitors at E13.5 and E15.5 were analyzed using flow cytometry and BrdU/EdU double labeling (Related to Fig 8). **(A and B)** Cell cycle flow cytometry analysis determining the percentage of cells in G0/G1, S, and G2/M phases in cortical progenitor cells from E13.5 **(A)** and E15.5 **(B)** *Ybx1* cKO and littermate control embryos. **(C)** Cell cycle kinetic analysis using BrdU/EdU double labeling. To estimate the cell cycle kinetic parameters, pregnant mice were injected with BrdU at *T* = 0 hrs to label all cells in the S phase at the beginning of the experiment. At *T* = 1.5 h, EdU was injected, and embryos were fixed after a short survival period of 0.5 h, which is sufficient to label the S phase cells at the end of the labeling period (S cells, co-labeled with BrdU and EdU). During the 1.5-h interval (*T*_*i*_), when cells are exposed to BrdU but not EdU, some cells from the initial S phase cohort exit the S phase and are thus labeled only with BrdU. These cells are referred to as the leaving fraction (L cells, labeled with BrdU only). **(D)** Immunostaining of Ki67, BrdU, and EdU in *Ybx1* cKO and control cortex at E14.5. Scale bars, 100 μm. **(E and F)** Calculation of cell cycle duration from experiments shown in **C** and **D**. Data are presented as box and whisker plots (*n* = 3 replicates): ****p = 2.08E−05 **(F)**; ns, not significant; by unpaired Student *t* test. The data underlying all the graphs shown in the figure are included in S1 Data.
(TIF)

S1 Table DEGs identified by RNA-seq analysis of *Ybx1* cKO embryos at E13.5 (Related to Fig 5). (XLSX)

S2 Table DEGs identified by RNA-seq analysis of *Ybx1* cKO embryos at E18.5 (Related to Fig 5). (XLSX)

S3 Table DEGs identified by RNA-seq analysis of *Ybx1* KD at E13.5 (Related to Fig 5). (XLSX)

S4 Table Ybx1 target mRNAs identified by anti-Ybx1 RIP-seq (Related to Fig 5). (XLSX)

S1 Data Raw data and statistical data analysis for all the relevant graphs. (XLSX)

## Abbreviations

AP: apical progenitors
BP: basal progenitor
BrdU: 5-bromo-2′-deoxyuridine
cleaved caspase-3: CC3
Ccnd2: cyclin D2
cKO: conditional knockout
CP: cortical plate
Cre: cre recombinase
DIG: digoxigenin
EdU: 5-ethynyl-2′-deoxyuridine
eEF1g: eukaryotic translation elongation factor 1 gamma
ISH: in situ hybridization
IUE: in utero electroporation
IPC: intermediate progenitor cell
IZ: intermediate zone
m^5^C: 5-methylcytosine
NSC: neural stem cell
RIP-seq: RNA immunoprecipitation-sequencing
RNA-seq: RNA-sequencing
RGC: radial glial cell
Rps5: ribosomal protein S5
Rpsa: ribosomal protein SA
SEM: standard error of the mean
shCtrl: control shRNA
sh*Ybx1*: shRNA against *Ybx1*
SVZ: subventricular zone
VZ: ventricular zone
Ybx1: Y-box binding protein 1
