# Supplementary figures and images for: The m5C reader Ybx1 regulates embryonic cortical neurogenesis by promoting progenitor cell cycle progression
Source: PLoS Biol. 2025 May 28;23(5):e3003175. doi: 10.1371/journal.pbio.3003175 (PMC12148234; doi:10.1371/journal.pbio.3003175)

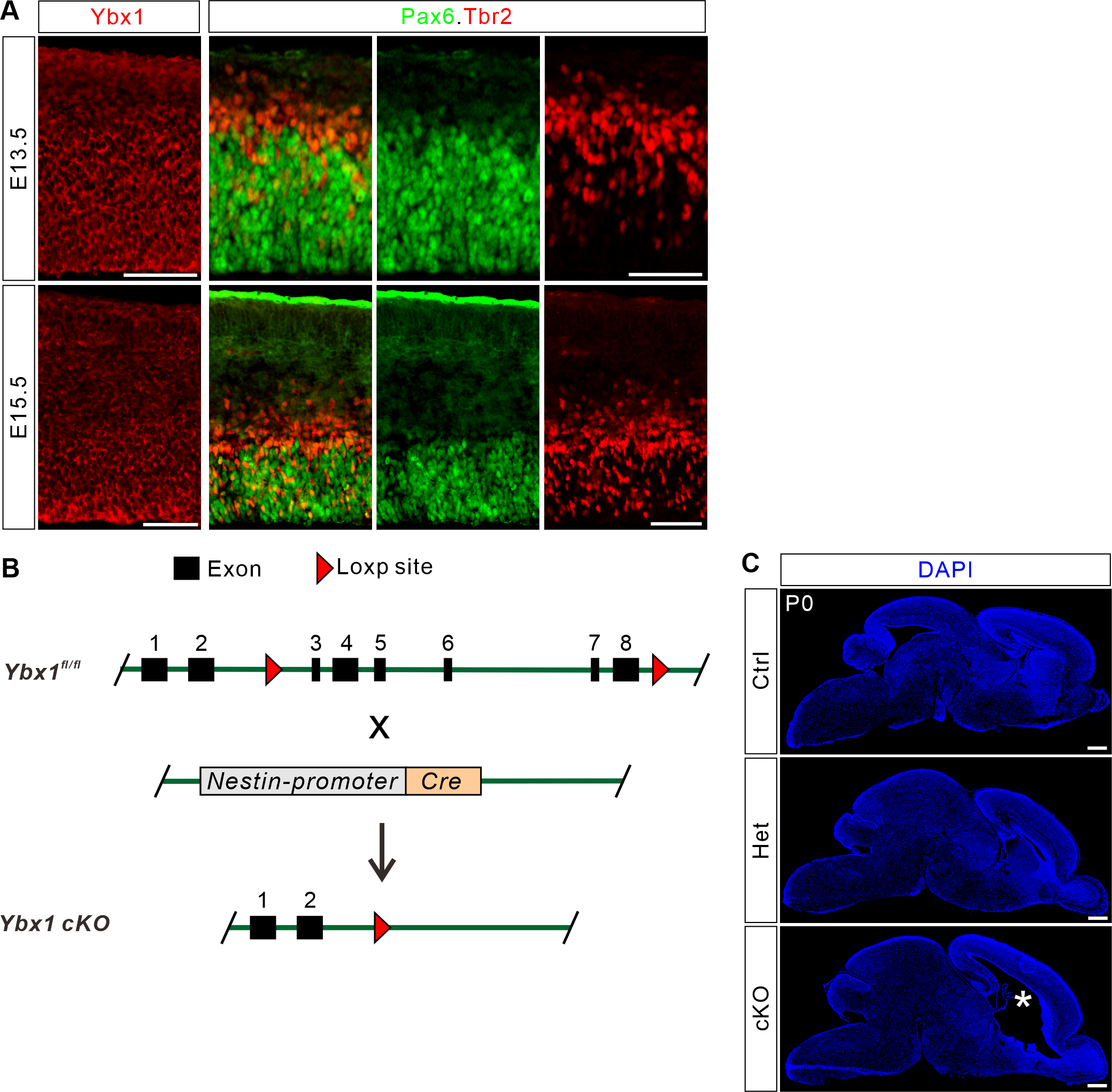

Supplement: S1 Fig — (A) Adjacent coronal brain sections from wild type (WT) mouse embryos at E13.5 and E15.5 were immunostained with antibodies against Ybx1, and Pax6 and Tbr2, respectively. Scale bars, 100 μm. (B) The exons 3 to 8 of the Ybx1 gene were deleted after Nestin-Cre-mediated recombination, resulting in the generation of Ybx1 conditional knockout (cKO) mice. (C) Representative images of P0 sagittal brain sections stained with DAPI. The asterisk indicates the enlarged ventricle in the cKO brain. Scale bar, 500 μm. (TIF) [file pbio.3003175.s001.tif]

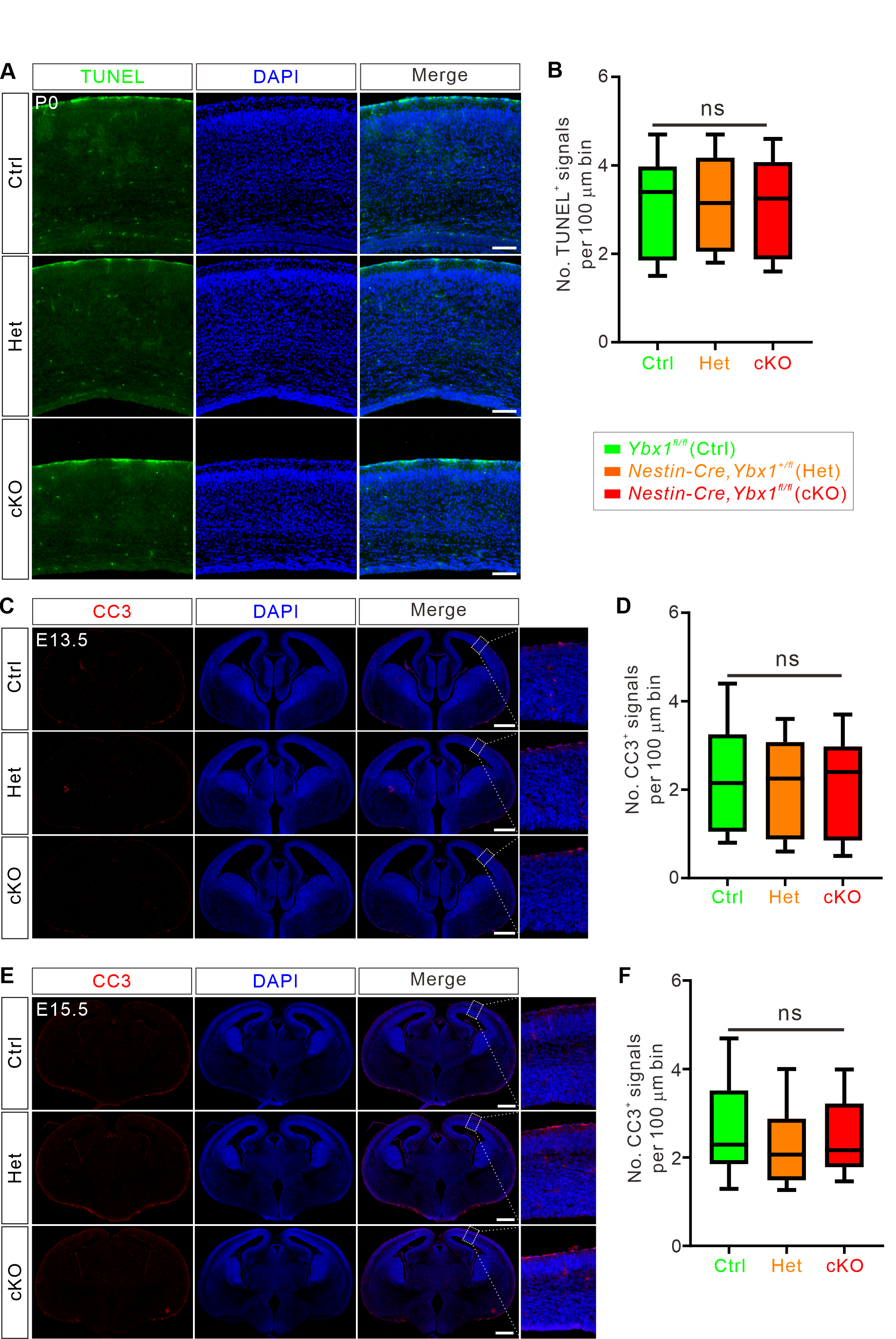

Supplement: S2 Fig — (A) Representative images of TUNEL staining in P0 cortex. Scale bar, 100 μm. (B) Quantification of TUNEL signals in P0 cortex is presented as a box and whisker plot. (C) Representative images of cleaved caspase-3 (CC3) staining in E13.5 cortex. Scale bar, 500 μm. (D) Quantification of CC3 signals in E13.5 cortex is presented as a box and whisker plot. (E) Representative images of CC3 staining in E15.5 cortex. Scale bar, 500 μm. (F) Quantification of CC3+ signals in E15.5 cortex is presented as a box and whisker plot. At least 3 mice were analyzed for each genotype. All analyses were performed by one-way ANOVA followed by Tukey’s multiple comparison test. ns, not significant. The data underlying all the graphs shown in the figure are included in S1 Data. (TIF) [file pbio.3003175.s002.tif]

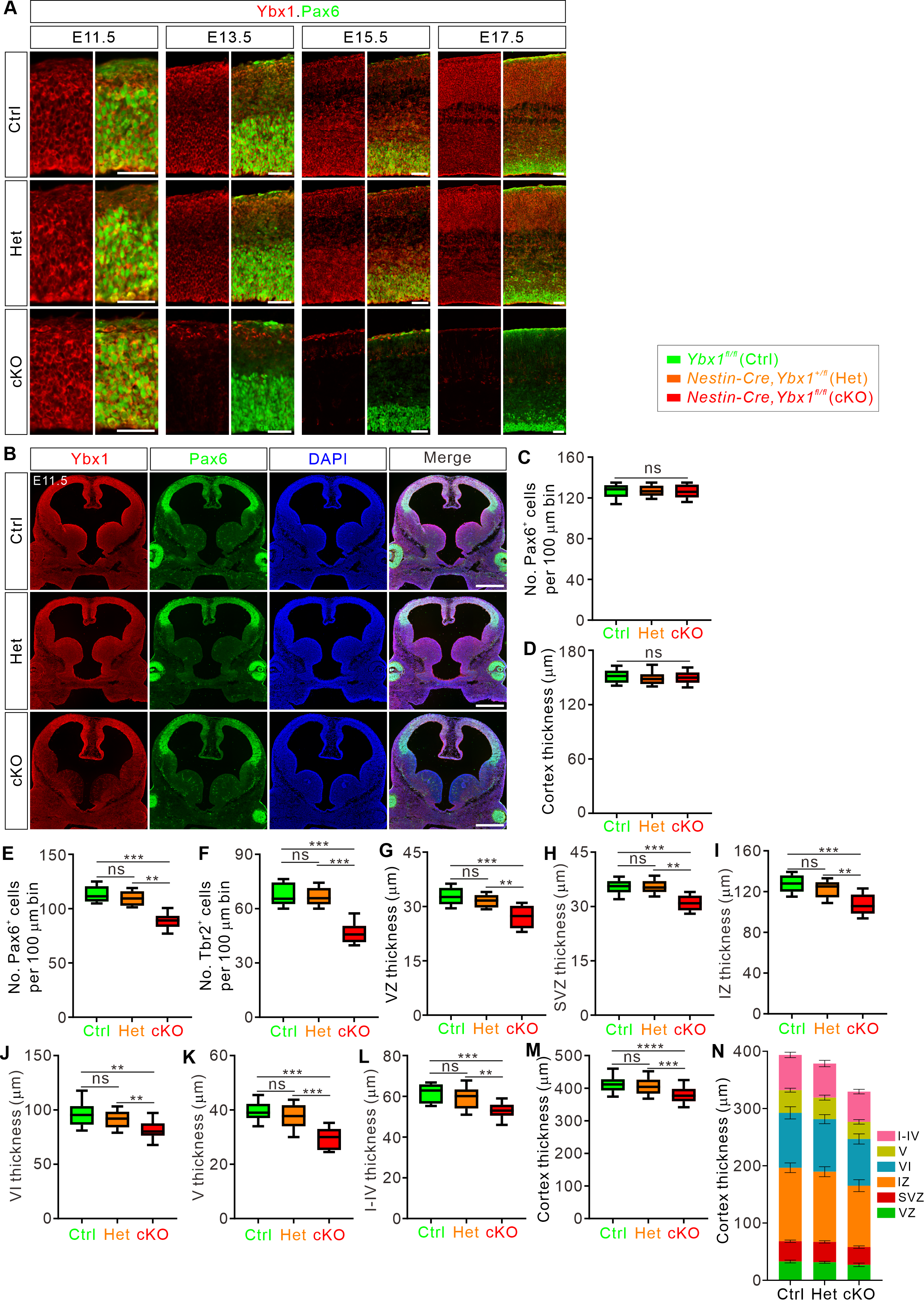

Supplement: S3 Fig — (A) Coronal brain sections at E11.5, E13.5, E15.5, and E17.5 were stained with antibodies against Ybx1 and Pax6. Representative cortical regions were shown. Scale bars, 100 μm. (B) Coronal brain sections at E11.5 were stained with antibodies against Ybx1 and Pax6. Scale bar, 500 μm. (C and D) Quantification of Pax6+ cell numbers (C) and whole cortical thickness (D) shown in (B). All statistical data are presented as box and whisker plots. ns, not significant. (E–M) Quantification of Pax6+ (E) and Tbr2+ (F) cell numbers, and cortical layer thicknesses (G to M) at E17.5. All statistical data are presented as box and whisker plots. For Pax6, Ctrl (n = 15 confocal fields) versus cKO (n = 14 confocal fields), ***p = 5.42E−04; Het (n = 15 confocal fields) versus cKO, **p = 0.0033. For Tbr2, Ctrl (n = 15 confocal fields) versus cKO (n = 14 confocal fields), ***p = 9.97E−04; Het (n = 15 confocal fields) versus cKO, ***p = 2.77E−04. For VZ thickness, Ctrl (n = 15 confocal fields) versus cKO (n = 14 confocal fields), ***p = 8.39E−04; Het (n = 15 confocal fields) versus cKO, **p = 0.0031. For SVZ thickness, Ctrl (n = 15 confocal fields) versus cKO (n = 14 confocal fields), ***p = 8.84E−04; Het (n = 15 confocal fields) versus cKO, **p = 0.0010. For IZ thickness, Ctrl (n = 18 confocal fields) versus cKO (n = 15 confocal fields), ***p = 2.46E−04; Het (n = 17 confocal fields) versus cKO, **p = 0.0036. For VI thickness, Ctrl (n = 18 confocal fields) versus cKO (n = 15 confocal fields), **p = 0.0016; Het (n = 17 confocal fields) versus cKO, **p = 0.0070. For V thickness, Ctrl (n = 18 confocal fields) versus cKO (n = 15 confocal fields), ***p = 3.89E−04; Het (n = 17 confocal fields) versus cKO, ***p = 2.82E−04. For I–IV thickness, Ctrl (n = 18 confocal fields) versus cKO (n = 15 confocal fields), ***p = 8.58E−04; Het (n = 17 confocal fields) versus cKO, **p = 0.0012. For whole cortical thickness, Ctrl (n = 18 confocal fields) versus cKO (n = 15 confocal fields), ****p = 4.07E−05; Het [file pbio.3003175.s003.tif]

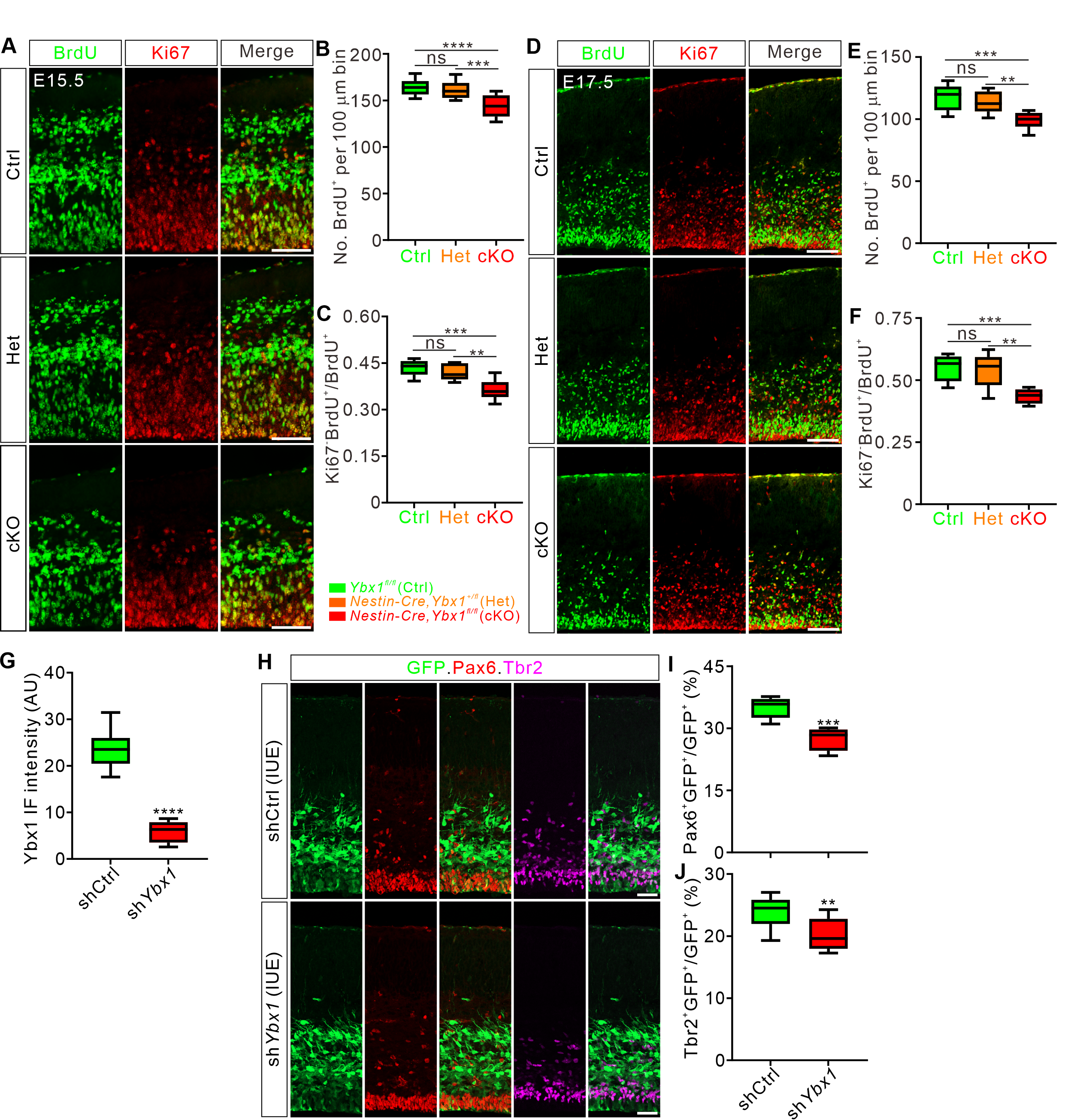

Supplement: S4 Fig — (A) Coronal brain sections at E15.5 were stained with antibodies recognizing BrdU and Ki67. Pregnant mothers received a BrdU pulse 24 h before embryo collection. Representative cortical regions were shown. Scale bar, 100 μm. (B and C) Quantification of BrdU+ cell numbers (B) and the percentage of cells exiting the cell cycle (C) at E15.5 shown in (A). All statistical data are presented as box and whisker plots. For BrdU+ cell numbers, Ctrl (n = 26 confocal fields) versus cKO (n = 23 confocal fields), ****p = 6.15E−05; Het (n = 21 confocal fields) versus cKO, ***p = 7.86E−04. For Ki67−BrdU+/BrdU+, Ctrl (n = 26 confocal fields) versus cKO (n = 23 confocal fields), ***p = 2.36E−04; Het (n = 21 confocal fields) versus cKO, **p = 0.0017. (D) Coronal brain sections at E17.5 were stained with antibodies recognizing BrdU and Ki67. Pregnant mothers received a BrdU pulse 24 h before embryo collection. Representative cortical regions were shown. Scale bar, 50 μm. (E and F) Quantification of BrdU+ cell numbers (E) and the percentage of cells exiting the cell cycle (F) at E17.5 shown in (D). All statistical data are presented as box and whisker plots. For BrdU+ cell numbers, Ctrl (n = 20 confocal fields) versus cKO (n = 19 confocal fields), ***p = 6.01E−04; Het (n = 19 confocal fields) versus cKO, **p = 0.0025. For Ki67−BrdU+/BrdU+, Ctrl (n = 20 confocal fields) versus cKO (n = 19 confocal fields), ***p = 2.55E−04; Het (n = 19 confocal fields) versus cKO, **p = 0.0015. (G) Quantification of Ybx1 immunofluorescence intensity in the cortex of shYbx1 embryos and controls. Data are presented as box and whisker plots: shCtrl (n = 29 confocal fields) versus shYbx1 (n = 27 confocal fields), ****p = 7.81E−09. (H) Immunostaining for GFP, Pax6, and Tbr2 on coronal sections of E16.5 mouse cortex after knockdown of Ybx1 using IUE. Representative cortical regions were shown. Scale bar, 50 μm. (I and J) Quantification of percentage of Pax6+GFP+/GFP+ (I) and Tbr2+GFP+/GFP+ (J) shown in (H). Da [file pbio.3003175.s004.tif]

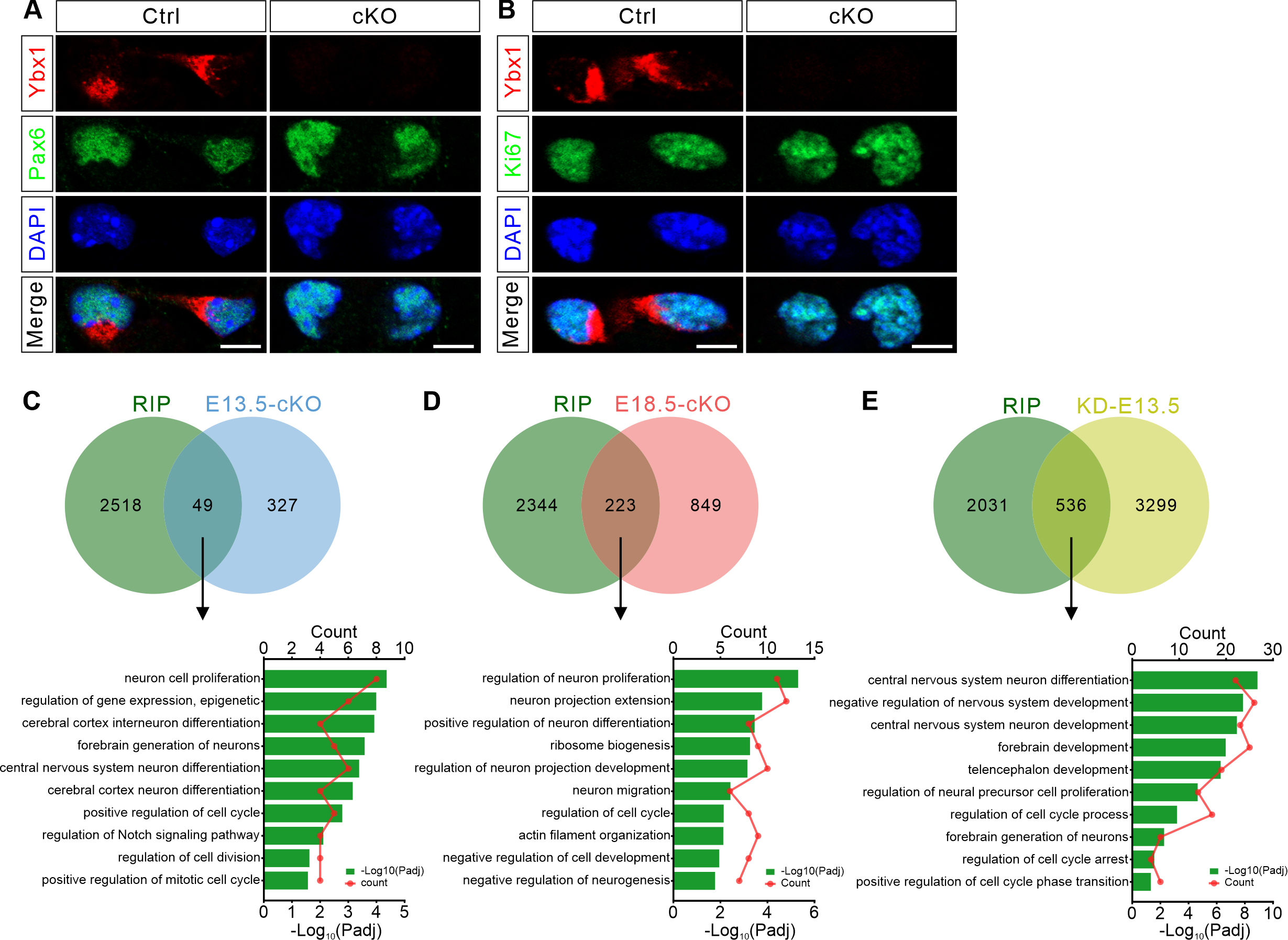

Supplement: S5 Fig — (A and B) Ybx1 immunostaining in cultured neural progenitor cells marked by Pax6 and Ki67 showed predominant signals in cytoplasm. The signals were absent in the cells from the Ybx1 cKO embryos, suggesting the specificity of Ybx1 immunofluorescence. Scale bars, 5 μm. (C–E) Venn diagrams showed the overlap of mRNAs identified by anti-Ybx1 RIP-seq with RNA-seq of Ybx1 cKO at E13.5 (C) and E18.5 (D), and Ybx1 KD at E13.5 (E). GO terms in biological processes were shown for these overlapped mRNAs. The data underlying all the graphs shown in the figure are included in S1 Data. (TIF) [file pbio.3003175.s005.tif]

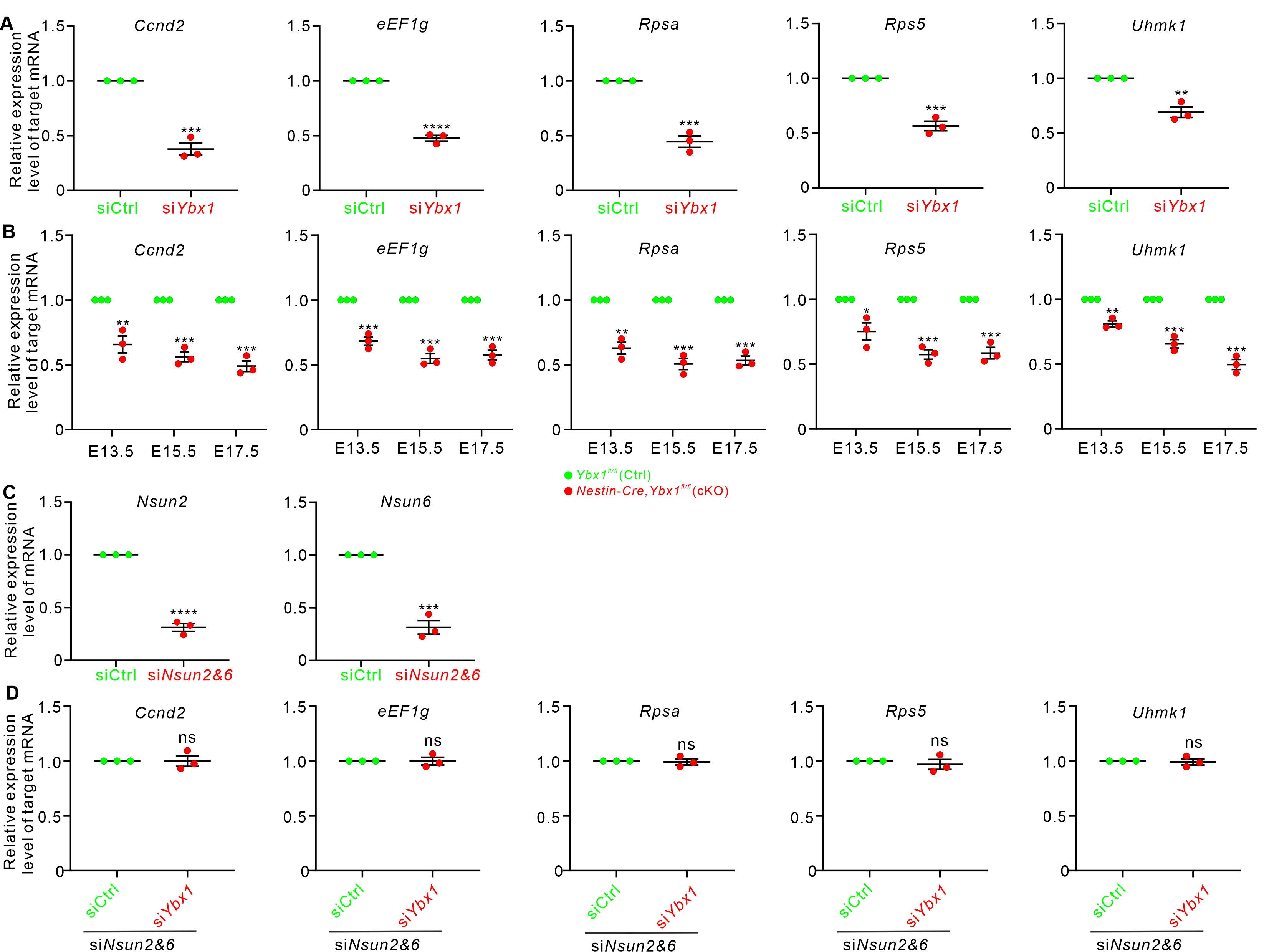

Supplement: S6 Fig — (A) RT-qPCR confirmed the decreased expression levels of Ybx1 target mRNAs in neural stem cells with Ybx1 knockdown using siRNA. Data are presented as mean ± SEM (n = 3 replicates): for Ccnd2, ***p = 0.00035; for eEF1g, ****p = 3.67E−05; for Rpsa, ***p = 0.00043; for Rps5, ***p = 0.00054; for Uhmk1, **p = 0.0031; by unpaired Student t test. (B) RT-qPCR confirmed the expression decreases of Ybx1 target mRNAs in the Ybx1 cKO cortices at E13.5, E15.5, and E17.5. Data are presented as mean ± SEM (n = 3 replicates): for Ccnd2, **p = 0.0063 (E13.5), ***p = 0.00032 (E15.5), ***p = 0.00023 (E17.5); for eEF1g, ***p = 0.00069 (E13.5), ***p = 0.00026 (E15.5), ***p = 0.00032 (E17.5); for Rpsa, **p = 0.0026 (E13.5), ***p = 0.00055 (E15.5), ***p = 0.00016 (E17.5); for Rps5, *p = 0.021 (E13.5), ***p = 0.00079 (E15.5), ***p = 0.00071 (E17.5); for Uhmk1, **p = 0.0012 (E13.5), ***p = 0.00048 (E15.5), ***p = 0.00019 (E17.5); by unpaired Student t test. (C) RT-qPCR validating knockdown efficiency of siRNAs against Nsun2 and Nsun6 in cultured cortical neural stem cells dissected from E13.5 mouse embryos. Data are presented as mean ± SEM: at least 3 replicates were analyzed for each experiment (n = 3); for Nsun2, ****p = 2.93E−05; for Nsun6, ***p = 0.00043; by unpaired Student t test. (D) Ybx1 could not regulate its target mRNA levels anymore after knockdown of m5C readers. Data are presented as mean ± SEM: at least 3 replicates were analyzed for each experiment (n = 3); ns, not significant; by unpaired Student t test. The data underlying all the graphs shown in the figure are included in S1 Data. (TIF) [file pbio.3003175.s006.tif]

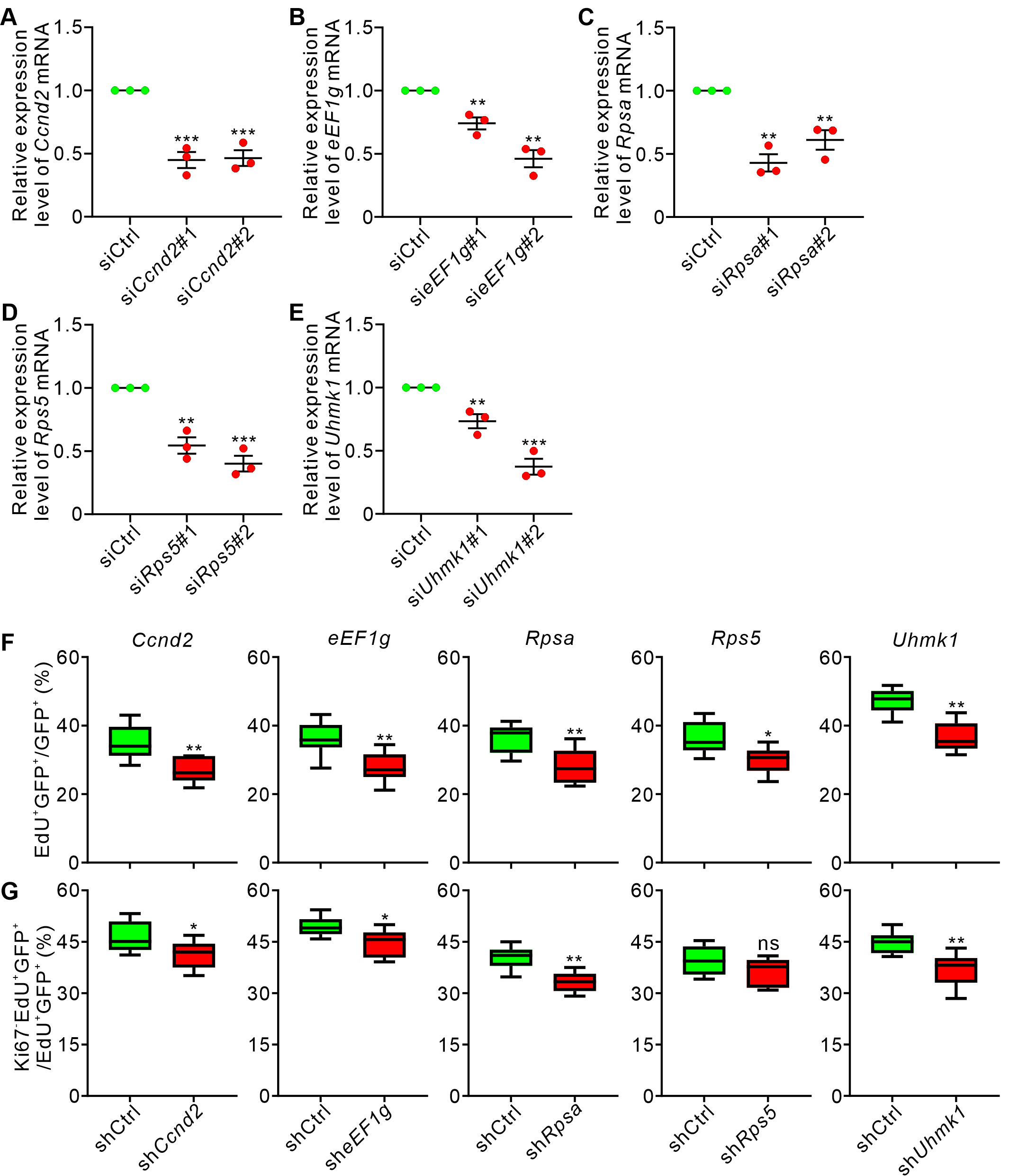

Supplement: S7 Fig — (A–E) RT-qPCR validated the knockdown efficiency of siRNAs targeting Ybx1 target mRNAs. Cortical neural stem cells dissected from E13.5 were cultured, transfected with siRNAs, and then RNA was extracted for RT-qPCR. Data are presented as mean ± SEM; at least 3 sets of replicates were analyzed for each experiment (n = 3). In A, siCcnd2#1 versus siCtrl, ***p = 0.00096; siCcnd2#2 versus siCtrl, ***p = 0.00099. In B, sieEF1g#1 versus siCtrl, **p = 0.0057; sieEF1g#2 versus siCtrl, **p = 0.0013. In C, siRpsa#1 versus siCtrl, **p = 0.0011; siRpsa#2 versus siCtrl, **p = 0.0074. In D, siRps5#1 versus siCtrl, **p = 0.0021; siRps5#2 versus siCtrl, ***p = 0.00064. In E, siUhmk1#1, **p = 0.0087; siUhmk1#1 versus siCtrl, ***p = 0.00057. All by unpaired Student t test. (F and G) Quantification of percentage of EdU+GFP+/GFP+ (F) and Ki67-EdU+/EdU+GFP+ (G) after knockdown of Ybx1 targets in the cortex using IUE of individual shRNA against each target. At least 3 embryos were analyzed for each condition. Data are presented as box and whisker plots. In F, shCtrl (n = 25 confocal fields) versus shCcnd2 (n = 23 confocal fields), **p = 0.0031; shCtrl (n = 20 confocal fields) versus sheEF1g (n = 21 confocal fields), **p = 0.0061; shCtrl (n = 27 confocal fields) versus shRpsa (n = 23 confocal fields), **p = 0.0052; shCtrl (n = 22 confocal fields) versus shRps5 (n = 24 confocal fields), *p = 0.030; shCtrl (n = 27 confocal fields) versus shUhmk1 (n = 26 confocal fields), **p = 0.0011. In G, shCtrl (n = 25 confocal fields) versus shCcnd2 (n = 23 confocal fields), *p = 0.013; shCtrl (n = 20 confocal fields) versus sheEF1g (n = 21 confocal fields), *p = 0.034; shCtrl (n = 27 confocal fields) versus shRpsa (n = 23 confocal fields), **p = 0.0049; shCtrl (n = 22 confocal fields) versus shRps5 (n = 24 confocal fields), p = 0.12; shCtrl (n = 27 confocal fields) versus shUhmk1 (n = 26 confocal fields), **p = 0.0031. All by unpaired Student t test. The data underlying all the graphs shown in the figur [file pbio.3003175.s007.tif]

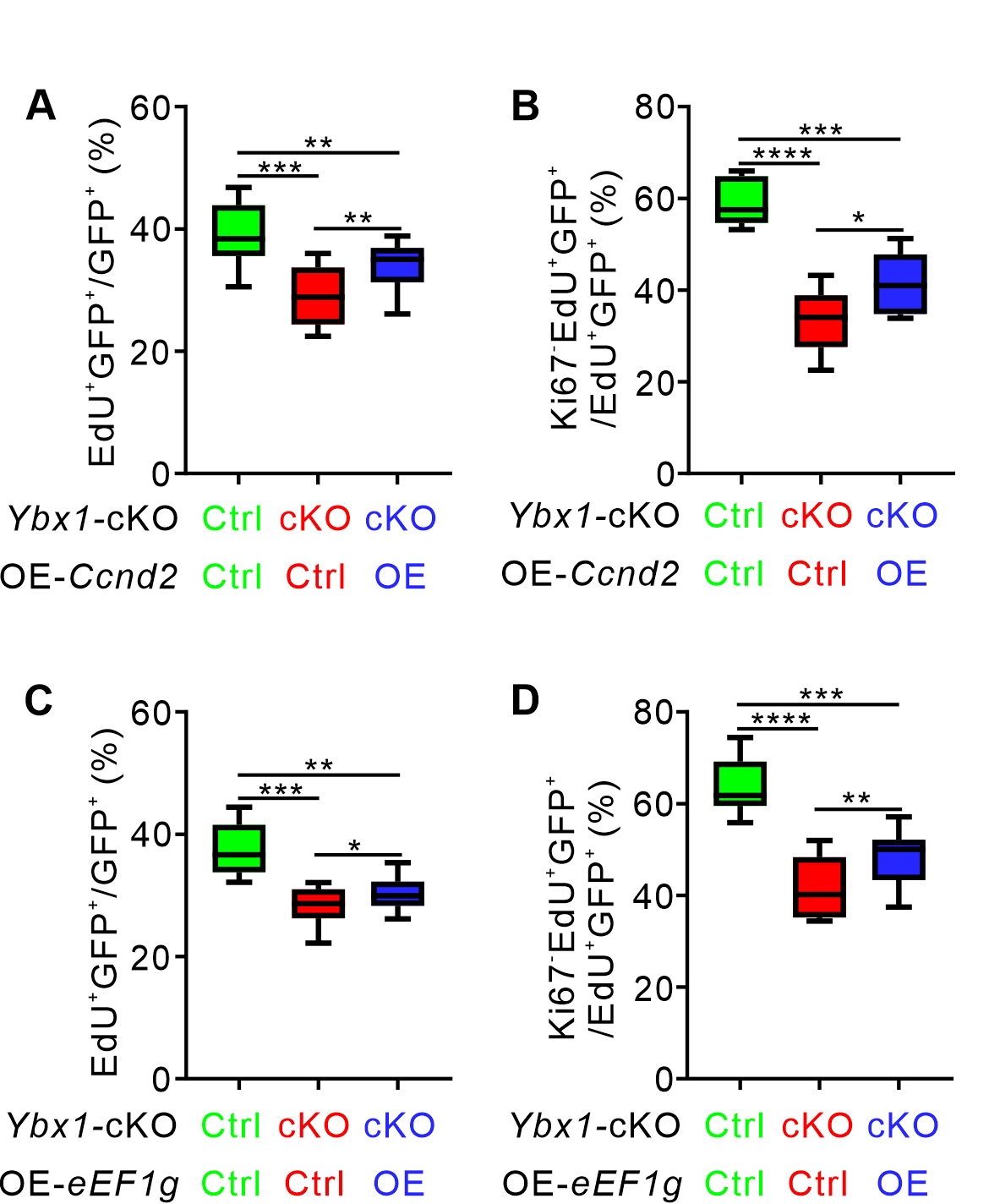

Supplement: S8 Fig — (A–D) Quantification of percentage of EdU+GFP+/GFP+ (A, C) and Ki67-EdU+GFP+/EdU+GFP+ (B, D) after overexpression of Ccnd2 (A, B) and eEF1g (C, D) in the cortex using IUE. Data are presented as box and whisker plots. In A, “Ctrl + OE-Ctrl” (n = 17 confocal fields) versus “cKO + OE-Ctrl” (n = 16 confocal fields), ***p = 1.57E−04; “cKO + OE-Ctrl” versus “cKO + OE-Cocktail” (n = 19 confocal fields), **p = 0.0052; “Ctrl + OE-Ctrl” versus “cKO + OE-Cocktail”, **p = 0.0046. In B, “Ctrl + OE-Ctrl” (n = 17 confocal fields) versus “cKO + OE-Ctrl” (n = 16 confocal fields), ****p = 5.67E−06; “cKO + OE-Ctrl” versus “cKO + OE-Cocktail” (n = 19 confocal fields), *p = 0.027; “Ctrl + OE-Ctrl” versus “cKO + OE-Cocktail”, ***p = 6.97E−04. In C, “Ctrl + OE-Ctrl” (n = 14 confocal fields) versus “cKO + OE-Ctrl” (n = 15 confocal fields), ***p = 2.64E−04; “cKO + OE-Ctrl” versus “cKO + OE-Cocktail” (n = 15 confocal fields), *p = 0.022; “Ctrl + OE-Ctrl” versus “cKO + OE-Cocktail”, **p = 0.0015. In D, “Ctrl + OE-Ctrl” (n = 14 confocal fields) versus “cKO + OE-Ctrl” (n = 15 confocal fields), ****p = 6.69E−05; “cKO + OE-Ctrl” versus “cKO + OE-Cocktail” (n = 15 confocal fields), **p = 0.0046; “Ctrl + OE-Ctrl” versus “cKO + OE-Cocktail”, ***P = 2.58E−04. Analyses were performed by one-way ANOVA followed by Tukey’s multiple comparison test. At least 3 embryos were analyzed for each genotype or condition. The data underlying all the graphs shown in the figure are included in S1 Data. (TIF) [file pbio.3003175.s008.tif]

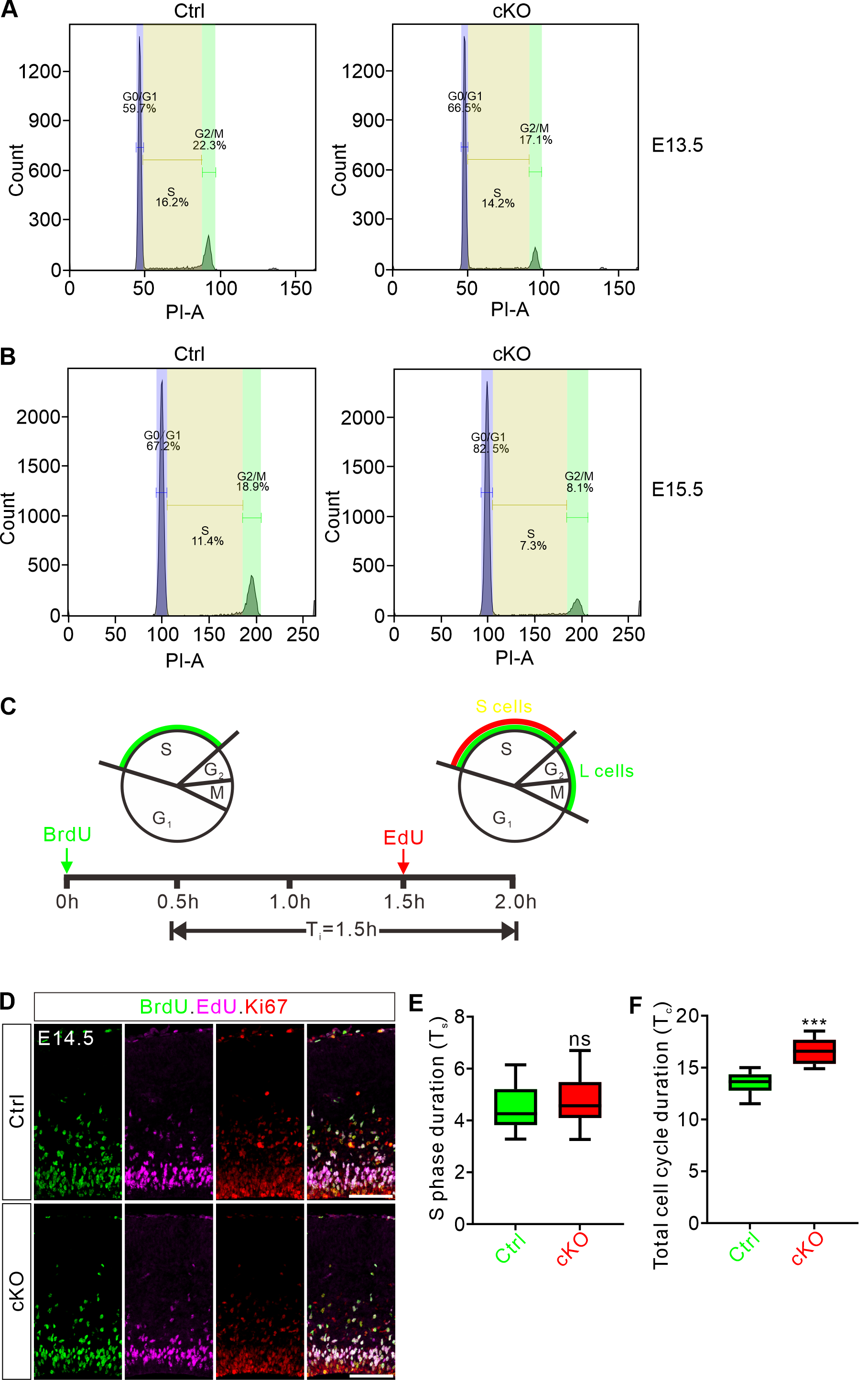

Supplement: S9 Fig — (A and B) Cell cycle flow cytometry analysis determining the percentage of cells in G0/G1, S, and G2/M phases in cortical progenitor cells from E13.5 (A) and E15.5 (B) Ybx1 cKO and littermate control embryos. (C) Cell cycle kinetic analysis using BrdU/EdU double labeling. To estimate the cell cycle kinetic parameters, pregnant mice were injected with BrdU at T = 0 hrs to label all cells in the S phase at the beginning of the experiment. At T = 1.5 h, EdU was injected, and embryos were fixed after a short survival period of 0.5 h, which is sufficient to label the S phase cells at the end of the labeling period (S cells, co-labeled with BrdU and EdU). During the 1.5-h interval (Ti), when cells are exposed to BrdU but not EdU, some cells from the initial S phase cohort exit the S phase and are thus labeled only with BrdU. These cells are referred to as the leaving fraction (L cells, labeled with BrdU only). (D) Immunostaining of Ki67, BrdU, and EdU in Ybx1 cKO and control cortex at E14.5. Scale bars, 100 μm. (E and F) Calculation of cell cycle duration from experiments shown in C and D. Data are presented as box and whisker plots (n = 3 replicates): ****p = 2.08E−05 (F); ns, not significant; by unpaired Student t test. The data underlying all the graphs shown in the figure are included in S1 Data. (TIF) [file pbio.3003175.s009.tif]
